# The duality of amyloid-β: its role in normal and Alzheimer’s disease states

**DOI:** 10.1186/s13041-024-01118-1

**Published:** 2024-07-17

**Authors:** Ali Azargoonjahromi

**Affiliations:** Shiraz University of Medical Sciences, Shiraz, Iran

**Keywords:** Alzheimer’s disease, Beta amyloid, Cognitive decline, Neuroprotection, Neurotoxicity, Neuroinflammation, Long-term potentiation

## Abstract

Alzheimer’s disease (AD) is a degenerative neurological condition that gradually impairs cognitive abilities, disrupts memory retention, and impedes daily functioning by impacting the cells of the brain. A key characteristic of AD is the accumulation of amyloid-beta (Aβ) plaques, which play pivotal roles in disease progression. These plaques initiate a cascade of events including neuroinflammation, synaptic dysfunction, tau pathology, oxidative stress, impaired protein clearance, mitochondrial dysfunction, and disrupted calcium homeostasis. Aβ accumulation is also closely associated with other hallmark features of AD, underscoring its significance. Aβ is generated through cleavage of the amyloid precursor protein (APP) and plays a dual role depending on its processing pathway. The non-amyloidogenic pathway reduces Aβ production and has neuroprotective and anti-inflammatory effects, whereas the amyloidogenic pathway leads to the production of Aβ peptides, including Aβ40 and Aβ42, which contribute to neurodegeneration and toxic effects in AD. Understanding the multifaceted role of Aβ, particularly in AD, is crucial for developing effective therapeutic strategies that target Aβ metabolism, aggregation, and clearance with the aim of mitigating the detrimental consequences of the disease. This review aims to explore the mechanisms and functions of Aβ under normal and abnormal conditions, particularly in AD, by examining both its beneficial and detrimental effects.

## Introduction

Alzheimer’s disease (AD) is a multifaceted neurological condition that involves the progressive degeneration of brain cells, resulting in cognitive decline, memory loss, and ultimately dementia [1]. As the leading cause of dementia, it accounts for roughly 60–70% of all dementia cases [2]. The disease typically progresses through stages, starting with mild memory lapses and leading to severe impairments in thinking, behavior, and the ability to carry out daily activities [3].

The pathological hallmarks of AD include amyloid-beta (Aβ) plaques and neurofibrillary tangles (NFTs), such as tau protein tangles [4], cholinergic dysfunction [5], glial cell activation [6], mitochondrial dysfunction [7], vascular abnormalities [8], calcium homeostasis [9], oxidative stress [10], and synaptic dysfunction [11]. These elements are associated with the degeneration of neurons, impede cognitive function, and require extensive medical attention [12, 13].

Of the various defining characteristics of AD, the accumulation of Aβ is regarded as a crucial pathological feature [14]. It is believed to occur early in the disease process and plays a pivotal role in the progression of AD [15]. Furthermore, Aβ accumulation has been observed to be associated with other hallmark features [16–20], underscoring its significance and the need to pay attention to its role in AD. The formation of Aβ plaques begins with the production of Aβ peptides through the sequential cleavage of the amyloid precursor protein (APP), a transmembrane protein found in many cells, including neurons [21].

Aβ exhibits a dual role contingent on the situation and the two processing pathways it undergoes: the non-amyloidogenic and amyloidogenic pathways. The non-amyloidogenic pathway diminishes the production and aggregation of Aβ peptides, imparting neuroprotection, fostering synaptic plasticity, and exerting anti-inflammatory effects [22, 23]. However, excessive emphasis on this pathway may disrupt the equilibrium of APP metabolism, resulting in accumulation of other fragments. Notably, the amyloidogenic pathway yields Aβ peptides, including Aβ40 and Aβ42, and the accumulation and aggregation of Aβ is closely linked to AD, provoking neurodegeneration and engendering toxic effects [24–27].

Understanding the multifaceted role of Aβ in different conditions, particularly AD, is of utmost importance. This understanding can help scientists develop effective therapeutic strategies that target Aβ metabolism, hinder aggregation, boost clearance mechanisms, and alleviate the detrimental consequences of AD. The objective of this study was to investigate the mechanisms and roles of Aβ in diverse conditions, with a specific emphasis on AD, by examining its positive and negative effects.

## Aβ production: physiological and pathological

Aβ, a small protein consisting of 39–43 amino acids, exists in different biophysical forms and can be generated by various cell types, including neurons, astrocytes, neuroblastoma cells, hepatoma cells, fibroblasts, and platelets [28–30]. Its presence in different species and cell types suggests that it plays a significant role in normal cell development and maintenance [31]. Among the cell types mentioned, neurons and smooth muscle cells demonstrate the highest levels of Aβ expression [32]. While the exact functions of Aβ in cell development and maintenance are not elaborated upon in the provided information, its widespread production and heightened expression in specific cell types imply its importance in cellular processes and homeostasis [33].

**Fig. 1:**
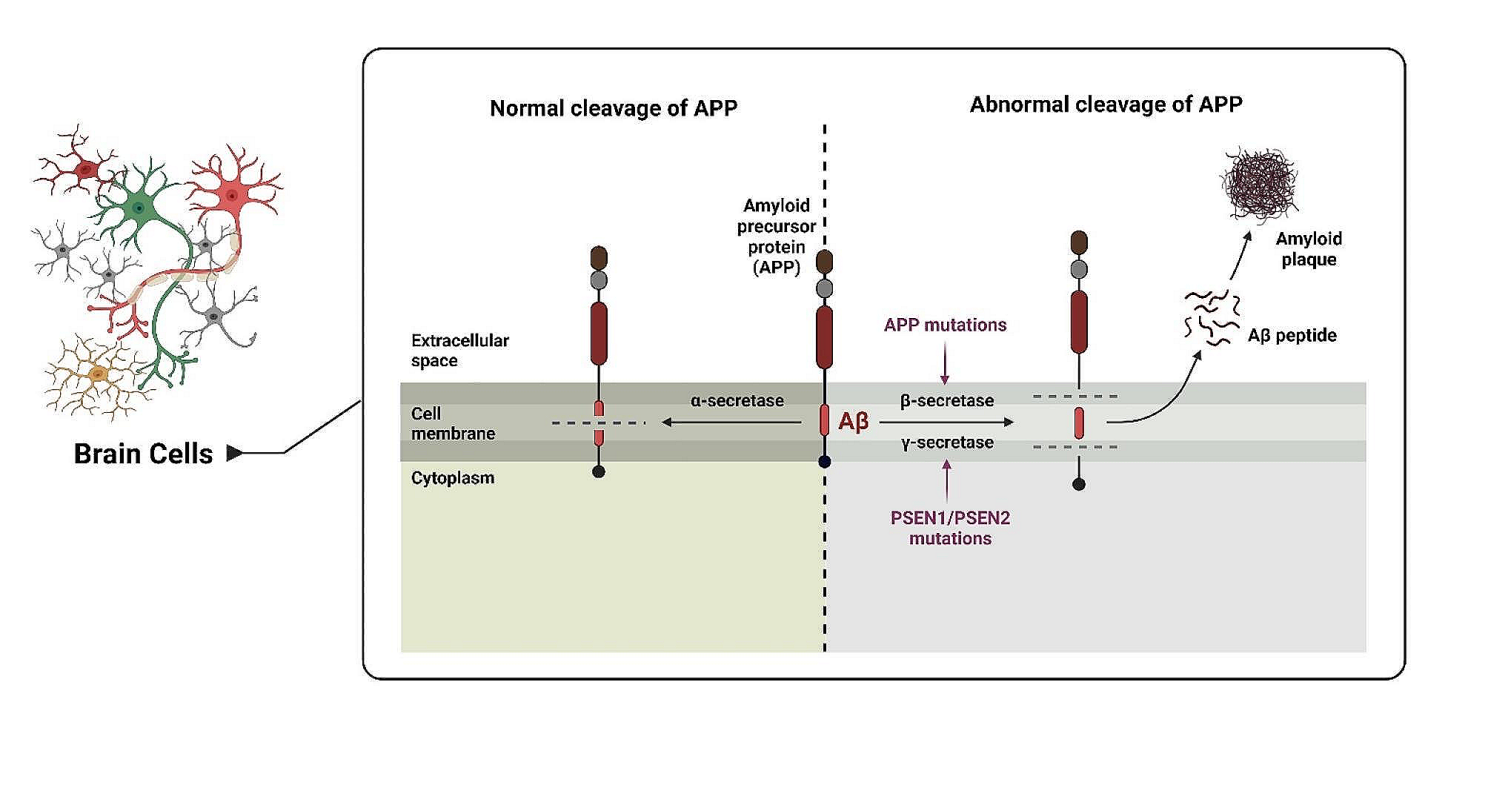
The non-amyloidogenic pathway plays a role in prevents the generation of Aβ by cleaving APP at α-secretase. In contrast, the amyloidogenic pathway involves β-secretase and γ-secretase, which are responsible for APP processing and contribute to the production of Aβ. Maintaining a balance between these pathways is important for the regulation of Aβ generation and its potential role in AD

The production of Aβ involves the enzymatic cleavage of APP; this cleavage can occur through two distinct pathways: The amyloidogenic pathway and the non-amyloidogenic pathway. In the amyloidogenic pathway, Aβ plaques are generated, while the non-amyloidogenic pathway does not produce Aβ plaques [24] (Fig. 1).

The non-amyloidogenic pathway serves as a natural mechanism to inhibit the production of Aβ. In this pathway, α-secretase recognizes APP within the Aβ domain, leading to the generation of soluble α-APP fragments (sAPPα) and C-terminal fragment α (CTFα, or C83). Subsequently, C83 is cleaved by γ-secretase, resulting in the formation of non-toxic P3 fragments and APP intracellular domain (AICD) fragments. This intricate process helps maintain a healthy equilibrium in APP processing [34, 35]. Two enzymes, ADAM10 and ADAM17 (also known as TACE), have been identified as α-secretases. ADAM10 is a member of the ADAM (a disintegrin and metalloproteinase) family, while ADAM17 is also known as a tumor necrosis factor-converting enzyme (TACE) [36]. These enzymes are capable of cleaving APP at the α-secretase cleavage site, thereby promoting the non-amyloidogenic pathway. In addition to ADAM10 and ADAM17, other proteases, such as ADAM9, ADAM12, ADAM19, and MDC9 have also been implicated in α-secretase activity [35].

In contrast, the amyloidogenic pathway entails a series of steps involving β-secretase and γ-secretase. Initially, β-secretase cleaves APP, resulting in the production of soluble β-APP fragments (sAPPβ) and C-terminal β fragments (CTFβ, or C99). Subsequently, γ-secretase cleaves C99, leading to the generation of AICD and Aβ [37]. The C99 fragment, generated by β-secretase, undergoes cleavage by γ-secretase within the cell membrane. This enzymatic process results in the release of the Aβ peptide, specifically the fragments Aβ40 and Aβ42 [38].

Most importantly, the accumulation of Aβ in AD can activate kinases such as GSK-3β, CDK5, and MAPKs, leading to abnormal phosphorylation of tau protein and its subsequent aggregation into NFTs. Additionally, disruption of phosphatases, enzymes that remove phosphate groups, can further contribute to tau hyperphosphorylation. The interaction between the amyloidogenic pathway and tau protein highlights the complex interplay between Aβ and tau pathology in AD, emphasizing the need for comprehensive therapeutic strategies targeting both aspects of the disease [39–41].

**Fig. 2:**
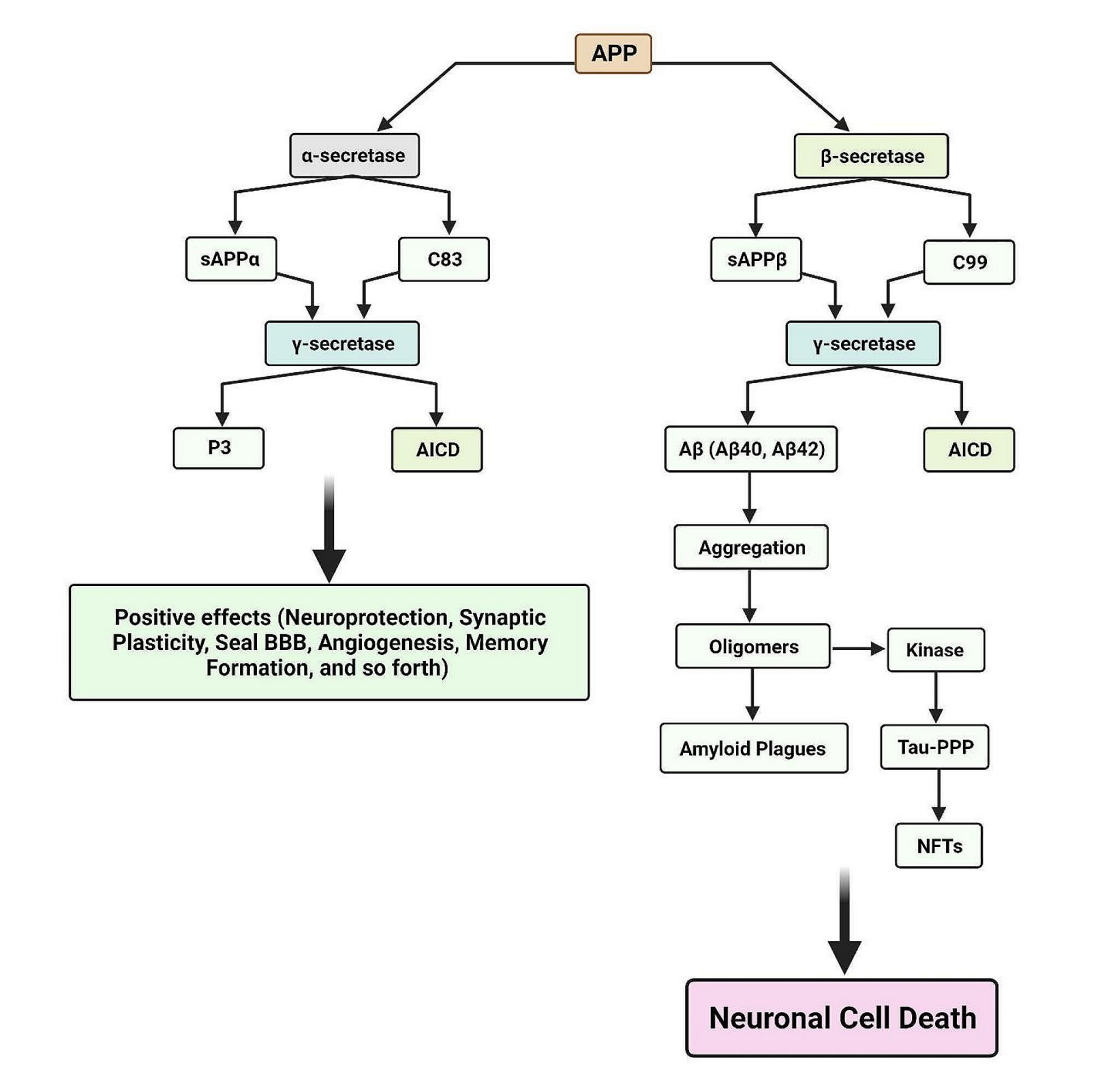
Two pathways delineate the fate of APP: the physiological route, where alpha-secretase cleaves APP to yield neuroprotective sAPPα and the benign C83 fragment, promoting neuronal health; and the pathophysiological cascade involving β-secretase and γ-secretase, producing toxic C99 (β-CTF) and subsequent Aβ peptides, notably Aβ42, leading to oligomerization, plaque formation, synaptic dysfunction, and neuronal damage. The former pathway emphasizes beneficial effects on neuronal function and signaling, whereas the latter links Aβ aggregates to neurotoxicity, oxidative stress, and inflammation, hallmarking AD progression

Notably, there is an imbalance between the activities of α-secretase and β-secretase in AD. β-Secretase activity increases, leading to enhanced cleavage of APP in the amyloidogenic pathway. As a consequence, there is an increased production of Aβ, particularly the more prone-to-aggregate Aβ42 fragment. The identification and cloning of the enzyme responsible for β-secretase cleavage resulted in the discovery of the beta-Site APP Cleaving enzyme (BACE). BACE is a membrane-bound aspartyl protease that initiates the amyloidogenic pathway by cleaving APP at the β-secretase site. The heightened activity of β-secretase, coupled with the aggregation-prone nature of Aβ42, leads to the accumulation of Aβ peptides, which subsequently aggregate to form amyloid plaques in the brain [42] (Fig. 2).

Notably, Aβ40 is more abundant than Aβ42 [43–45], yet in the formation of amyloid plaques, Aβ42 is the main component [46–49]. Aβ plaques trigger a series of downstream events that contribute to neurodegeneration in AD. The most important of these include neuroinflammation, synaptic dysfunction, tau pathology, oxidative stress, impaired protein clearance, mitochondrial dysfunction, and disruption of calcium homeostasis [50–52]. These interconnected processes further exacerbate neuronal damage, leading to the cognitive impairment and progressive decline observed in AD [53].

## Positive effects of the Aβ

The potential protective effects of Aβ in brain cells have received limited attention and have often been disregarded. Nonetheless, emerging evidence indicates that under specific circumstances, Aβ may display protective, trophic, or antioxidative physiological effects [54, 55]. This suggests that the physiological role of Aβ in the nervous system may be altered under certain conditions, potentially leading to toxic pathological effects that will be discussed in the ensuing headings of this article.

### Aβ as an antioxidant

Aβ is a peptide containing two crucial sites responsible for its redox function. The first site plays a significant role in binding transition metals, effectively reducing their participation in oxidative damage. The second site, located in the lipophilic portion at the C-terminus of the peptide, acts as a trap for free radicals and participates in metal reduction, thereby exerting antioxidative and pro-oxidative effects. Aβ demonstrates a higher affinity for copper (Cu) than for iron (Fe) when it comes to metal binding, and its binding capacity matches that of chelating agents such as EDTA. The slow reduction of transition metals by Aβ suggests that it functions as an endogenous scavenger, gradually neutralizing these metals [54]. Cell studies have confirmed the protective and antioxidative effects of Aβ, reducing apoptotic death in neuronal cultures and decreasing lipoprotein oxidation in cerebrospinal fluid and blood plasma, possibly due to its chelating ability over metals, particularly Cu [55–57].

In the context of AD, cerebrospinal fluid (CSF) possesses a property that helps protect against oxidative damage, which is crucial in the development of the disease [58]. This property is closely associated with the level of Aβ1–42, a specific form of Aβ protein, in the CSF. Aβ1–42 have a higher affinity for binding to metals, enabling it to chelate or bind to metals effectively. This metal-chelating ability is believed to contribute to its superior antioxidative role in the CSF by preventing oxidative damage caused by metals. The antioxidative aspects of CSF correlate more strongly with Aβ1–42 levels than with ascorbate levels, which is an important antioxidant in the CSF [59]. Overall, these findings suggest that Aβ1–42 and its metal-chelating function play a significant role in the ability of the CSF to protect against oxidative damage, potentially impacting the progression of AD.

Notably, cells overexpressing Aβ exhibited lower ROS production and reduced susceptibility to metal damage. In cortical neuron cultures, inhibiting β- and γ-secretases or aggregating Aβ antibodies reduces cell viability; however, this effect is completely reversed by adding Aβ1–40 [60]. In a relevant study involving neural stem cells (NSCs), it was found that oligomers of Aβ1–42, at a concentration of 1 µM, promoted the survival and differentiation of striatal and hippocampal NSCs. Notably, this beneficial effect was not observed when Aβ1–40 or Aβ25–35 was administered, nor with the fibrillar forms of these peptides [61]. In initial in vivo studies conducted in rats, the implantation of Aβ in the hippocampus did not result in any observed neurotoxic effects from a morphological standpoint [62]. Furthermore, subsequent studies investigating the long-term administration of different Aβ peptides (1–40, 1–38, 25–35) at various doses (ranging from 5 ng to 10 µg) in the cortex and hippocampus of adult rats did not reveal any discernible toxic effects when compared to the control group [63].

Interestingly, the direct administration of low concentrations of Aβ into the brains of young animals, including monkeys and rodents, has not been observed to cause neuronal damage. However, in older animals, Aβ can impact neurons. The underlying reasons for this age-dependent disparity are not yet well comprehended, although it is speculated that higher levels of free metals in the brains of older animals or a decline in natural antioxidative defenses associated with aging may contribute to this phenomenon. Intriguingly, Aβ may exhibit antioxidant properties [64]. In experimental models of mitochondrial dysfunction caused by inhibitors of mitochondrial complexes I and III, such as rotenone and antimycin, there was a notable rise in oxidative stress and a significant increase in Aβ production. Importantly, the use of antioxidants has been shown to reverse this heightened Aβ production, highlighting their potential in mitigating the effects of mitochondrial dysfunction on Aβ accumulation [65]. Prior research has primarily emphasized the antioxidant properties of nonfibrillar Aβ. Nonetheless, a recent study put forth the notion that even in its aggregated form, within the concentration range of 2 to 20 µM, Aβ can diminish the generation of hydroxyl radicals and hydrogen peroxide in synthetic nonbiological systems. Furthermore, it has the potential to safeguard proteins and lipids against oxidation in isolated mitochondria obtained from rat brains [66].

As shown above, some studies suggest that Aβ’s primary physiological function is as an endogenous antioxidant, causing increased production in normal aging. This leads to oxidative stress, resulting in a chronic redox imbalance in AD, where overproduction becomes toxic.

### Aβ as a neuroprotector

In a study conducted by Giuffrida et al., synthetic Aβ 1–42 monomers exhibited neuroprotective effects in neuronal culture. When administered at a concentration of 0.1 µM, these monomers prevented cell death induced by the deprivation of trophic factors such as insulin. Additionally, at concentrations ranging from 30 to 100 nM, they provided protection against the excitotoxic effects induced by NMDA, both before and after the excitotoxic stimulus. Notably, this protective effect is associated with activation of the phosphatidylinositol 3-kinase (PI-3 K) pathway. Interestingly, when Aβ 1–42 monomers with the Arctic (E22G) mutation were used, no neuroprotective effects were observed. This suggests that the altered peptide conformation resulting from this mutation significantly affects the ability of Aβ to exert its protective effects [67]. Another study provided further confirmation that nonfibrillar Aβ 1–42, at concentrations of up to 1 µM, has the ability to decrease cell death and inhibit the entry of intracellular calcium triggered by NMDA receptor activation. However, this protective effect was not observed when AMPA receptor activation was induced. These findings suggest that the neuroprotective properties of nonfibrillar Aβ 1–42 are specific to NMDA receptor-mediated processes and may not extend to AMPA receptor-mediated mechanisms [68].

Interestingly, in a study, brain slice cultures from a mouse model of AD were treated with N-terminal Aβ fragments (N-Aβcore) to investigate their effects on astrogliosis, microgliosis, and synaptic alterations. The researchers also examined the impact of N-terminal Aβ fragments on neuron/glial cultures and a microglial cell line exposed to pathological concentrations of Aβ. The results demonstrated that N-terminal Aβ fragments had several beneficial effects, including mitigating Aβ-induced astrogliosis and microgliosis, protecting against oxidative stress, mitochondrial dysfunction, and apoptosis in astrocytes and microglia, reducing the expression and release of proinflammatory mediators in activated microglial cells, and rescuing Aβ-induced synaptic loss. These findings emphasize the protective role of N-terminal Aβ fragments in alleviating neuroinflammation and synaptic damage associated with the development of AD [69].

### Aβ as a memory consolidator

In initial electrophysiological studies performed on hippocampal slices, it was observed that Aβ at concentrations in the nanomolar range (100–200 nM) facilitated long-term potentiation (LTP) and increased synaptic currents mediated by NMDA receptors (NMDAr), but had no impact on currents mediated by AMPA receptors (AMPAr) [70, 71]. Subsequent investigations using hippocampal slices revealed that administration of Aβ 1–40 at a concentration of 83 nM restored the impaired ability to generate LTP resulting from prolonged incubation of the slices. Notably, this restorative effect was reversed when the cholesterol synthesis was inhibited. These findings led the authors to propose that Aβ 1–40 may enhance the dynamics and availability of membrane cholesterol, thereby contributing to its facilitatory effects on synaptic plasticity [72].

Accordingly, Aβ modulates these glutamatergic receptors, facilitating LTP and contextual fear memories, while high picomolar concentrations disrupt glutamate clearance, resulting in aberrant activation of NMDA receptors and synaptic dysfunction, underscoring the intricate nature of Aβ’s impact on glutamatergic signaling [73].

Another study confirmed that applying low concentrations of Aβ 1–42 (200 pM) to hippocampal slices enhances LTP, which is associated with improved reference memory and context fear memory in vivo [74, 75]. The study also found that the positive effect of Aβ on synaptic plasticity may be mediated by α7 receptors, as the administration of α7-nicotinic antagonists suppresses LTP [74]. In an in vivo study conducted on rats, it was demonstrated that sequestering endogenous Aβ using a monoclonal antibody against the Aβ ectodomain had a significant impact on the retention of short- and long-term memory in an inhibitory avoidance task. The antibody was infused into the hippocampus prior to training. Interestingly, this effect was not observed when the antibody was administered after the training sessions. These findings were consistent with the effects observed when mecamylamine, a nicotinic cholinergic receptor antagonist, was administered. Notably, the study also revealed that learning impairment could be reversed by administering exogenous human Aβ 1–42 directly into the hippocampus after training. This finding highlights the role of Aβ in memory consolidation [76].

Likewise, in another study, both in vitro and in vivo experiments were conducted to investigate the effects of simultaneous administration of anti-Aβ antibodies and interference RNA on various cognitive measures such as LTP, spatial reference memory, and contextual fear conditioning. The results showed that this combination treatment altered these cognitive functions. However, these effects could be reversed by administering Aβ 1–42 at specific concentrations (200–300 pM). Notably, the study also found that the positive effects of Aβ 1–42 were absent in mice that lacked the α7-nicotinic cholinergic receptor. This suggests that the α7-nicotinic cholinergic receptor may be involved in mediating the beneficial effects of Aβ 1–42 on cognitive function [75].

Nonetheless, in a dose-response study, the hormetic effect of Aβ 1–42 on LTP and spatial memory in the Morris maze was examined. The findings revealed that Aβ 1–42 had stimulatory effects within a specific dose range of 2 pM to 2 nM. However, at higher concentrations ranging from 2 to 20 µM, negative effects were observed. These results suggest that the effects of Aβ can be ambivalent and dependent on the dose administered. Furthermore, the study highlights that the positive effects of Aβ may be attributed to its direct interaction with the α7-cholinergic nicotinic receptors [77].

Aβ has the potential to enhance LTP by increasing the release of acetylcholine, a neurotransmitter involved in learning and memory [78], into the synaptic cleft. Moreover, Aβ may augment synaptic strengthening by increasing the likelihood of depolarization of postsynaptic neurons. Experimental studies administering low concentrations of Aβ into the hippocampus of mice have revealed improved memory retention in two memory tasks [79]. Additionally, these studies demonstrated elevated acetylcholine production specifically in the hippocampus, indicating a potential connection between Aβ, acetylcholine, and memory enhancement [79, 80]. These findings provide valuable insights into the intricate role of Aβ in synaptic function and memory processes, contributing to our understanding of neurodegenerative disorders, such as AD.

At varying concentrations, Aβ exerts contrasting effects on the α7-nicotinic acetylcholine receptors. At picomolar concentrations, Aβ directly activates these receptors, whereas at nanomolar concentrations, it blocks and deactivates these receptors. Notably, studies have shown that picomolar concentrations of Aβ42 enhance LTP and facilitate memory consolidation in mice. Conversely, nanomolar concentrations of Aβ impair memory functions. The effectiveness of Aβ-mediated memory enhancement is contingent on the presence of α7-nicotinic acetylcholine receptors. These findings emphasize the intricate and concentration-dependent interplay between Aβ, α7-nicotinic acetylcholine receptors, and memory processes [81–83].

### Aβ as a regulator of blood brain barrier (BBB) and angiogenesis

According to the vascular hypothesis, alterations in the cerebral vasculature system, including disruption of the BBB and angiogenesis, may contribute to the development of AD [84, 85]. However, it has been observed that non-pathological Aβ peptides can regulate angiogenesis [86] and potentially protect against BBB leakages [33, 87, 88]. Research indicates that Aβ peptides may function as a protective seal, preserving the integrity of the BBB and preventing cerebrovascular changes [87, 89].

A study highlighted the potential role of Aβ in maintaining the integrity of the cerebral vasculature. When Aβ deposits were cleared through immunotherapy in a mouse model with cerebral amyloid angiopathy, there were instances of cerebral microhemorrhage, suggesting that Aβ may play a role in preventing vascular leakage [90]. Additionally, in another experiment, non-AD mice were exposed to *Chlamydia pneumoniae*, a bacterium known to disrupt the BBB, which surprisingly led to the deposition of Aβ in the brain [91, 92]. These findings imply that Aβ production may be triggered as a protective response when the BBB is disturbed, thus serving as a sealant to counteract the effects of such disruptions. This suggests a potential relationship between Aβ levels, vascular integrity, and BBB maintenance.

Aβ exerts contrasting effects on angiogenesis in a dose-dependent manner. Specifically, at nanomolar concentrations, Aβ promotes endothelial cell proliferation and angiogenesis, whereas at micromolar concentrations, it inhibits proliferation, induces morphological changes, and causes cell death [93, 94]. Notably, Aβ peptides demonstrate functional similarity to fibroblast growth factor-2 and exhibit synergistic activity in enhancing angiogenesis [95, 96]. Furthermore, studies utilizing zebrafish models have revealed that Aβ enhances blood vessel branching, as evidenced by increased branching in response to human monomeric Aβ42 [93]. Conversely, zebrafish embryos lacking APP exhibit vascular abnormalities, which can be partially restored by Aβ injection [97]. Additionally, the inhibition of β-secretase, an enzyme involved in Aβ production, leads to vascular defects. However, further investigation of the effects of picomolar concentrations of Aβ on angiogenesis would contribute to a more comprehensive understanding of its physiological function [98].

Most importantly, Aβ peptides exert a dose- and conformation-dependent influence on angiogenesis. Specifically, the oligomeric form of Aβ peptides displays anti-angiogenic activity, whereas the fibrillar forms lack this effect [99]. The amino acid sequence HHQKLVFF has been identified as the component responsible for this anti-angiogenic activity [100]. Conversely, Aβ35–42, which contains a pro-angiogenic motif, exhibits a pro-angiogenic effect [100, 101]. When incubated with human umbilical vein endothelial cells, Aβ35–42 promotes increased formation of endothelial tip cells, further supporting its proangiogenic properties [102].

## AD’s pathological features related to Aβ plaques

In AD, abnormalities in APP processing lead to the generation of excess Aβ, a protein fragment derived from APP. This abnormal processing involves enzymes called secretases, with β-secretase cleaving APP to produce sAPPβ [103] and a longer fragment called C99. Subsequently, C99 is further cleaved by γ-secretase, resulting in the release of Aβ peptides [104], including the more common form, Aβ40, and the more toxic form, Aβ42 [105–107].

Notably, the length of Aβ peptides can vary; Aβ peptides ranging from 38 to 43 amino acids have been observed, and different forms of Aβ have varying degrees of amyloidogenicity. For instance, Aβ1–42 and Aβ3–40 are considered more amyloidogenic, meaning that they have a higher tendency to form amyloid plaques associated with AD pathology. In contrast, Aβ1–40 and Aβ1–38 are less amyloidogenic [108, 109]. APP processing and subsequent Aβ production mainly occur within the endosomal compartment and the trans-Golgi network of cells. This suggests that these subcellular localizations are where most Aβ is generated before being secreted through exocytosis [109]. In addition to extracellular deposition, intracellular accumulation of Aβ has been observed in both animal models of AD and human patients [108–110]. However, the significance of intracellular Aβ remains uncertain and is an area of ongoing research.

### Aβ and proteases

Aβ can undergo cleavage by various proteases, including insulin-degrading enzyme [111], neprilysin [112], BACE1 [113], and cathepsin B (specifically for Aβ1–42) [114]. Insulin-degrading enzyme (IDE), neprilysin, and cathepsin B play roles in rendering Aβ non-amyloidogenic, meaning they help prevent the aggregation and formation of amyloid plaques. The importance of Aβ11–40, which is generated by BACE1 cleavage at the β’ site, is still not fully understood. Other modifications to Aβ can occur as well. For example, the formation of pyroglutamate at the amino-terminal glutamic acid residue leads to the generation of truncated pyroglutamate Aβ3–40/42, which has a high propensity to form amyloid plaques. Inhibition of the enzyme responsible for pyroglutamate formation has shown promise in reducing amyloidosis and improving cognition in mouse models of AD [115].

#### Insulin-degrading enzyme (IDE)

IDE, also known as Insulysin, is a highly conserved zinc metalloprotease that is present in various tissues, including the brain [116]. While its primary function is to degrade insulin, IDE also plays a crucial role in the degradation of other peptides, including Aβ. IDE recognizes Aβ peptides and enzymatically cleaves them at specific sites, resulting in the breakdown of Aβ into smaller fragments, helps prevent the accumulation of Aβ and the subsequent formation of plaques in the brain. The degradation of Aβ by IDE is significant in maintaining the balance between Aβ production and clearance [117, 118].

However, in AD, IDE function may become impaired or overwhelmed as a result of oxidative stress and inflammation. For instance, In AD, chronic inflammation is observed in the brain, and inflammatory molecules can alter IDE expression or activity levels [119]. Pro-inflammatory cytokines, such as interleukin-1β (IL-1β) and tumor necrosis factor-alpha (TNF-α), have been shown to suppress IDE expression and activity, leading to reduced Aβ degradation [120, 121]. Moreover, genetic variations in the IDE gene have also been associated with altered IDE function and an increased risk of AD [122, 123]. Indeed, when IDE is unable to efficiently degrade Aβ, there is an increase in Aβ levels in the brain; the accumulation of Aβ can promote the formation of amyloid plaques, which can trigger a cascade of events leading to neuroinflammation, neuronal dysfunction, and ultimately, cognitive decline in AD.

#### Neprilysin

Neprilysin, which is a type II integral membrane protein belonging to the zinc metalloendopeptidase family, has a large extracellular domain responsible for substrate binding and enzymatic activity [124]. Neprilysin can cleave various peptides, including neuropeptides, hormones, and Aβ peptides [125].

In AD, neprilysin plays a critical role in the degradation and clearance of Aβ peptides in the brain [126, 127]. To clarify, neprilysin targets Aβ at specific sites, breaking it down into smaller fragments that are more soluble. These smaller fragments can be efficiently cleared from the brain through enzymatic degradation or clearance mechanisms like the BBB [128–131].

#### Cathepsin B

Cathepsin B, which is a lysosomal cysteine protease, plays a role in Aβ metabolism and AD. It is primarily found in lysosomes and can cleave Aβ1–42, a form of Aβ peptide, at specific sites, generating smaller fragments [132].

Cathepsin B, through its cleavage of Aβ peptides, can influence their aggregation and neurotoxicity, with increased activity potentially enhancing Aβ degradation and clearance; however, impaired cathepsin B function or lysosomal dysfunction can compromise Aβ clearance, leading to peptide accumulation and amyloid plaque formation. Factors such as oxidative stress, inflammation, and changes in the lysosomal environment regulate cathepsin B activity, which not only affects Aβ metabolism but also contributes to neurodegeneration by damaging neurons and disrupting lysosomal function [114, 133]. Further research is needed to fully understand cathepsin B’s role in Aβ metabolism and its potential as a therapeutic target for AD.

### Aβ sequence alteration

Certain modifications in the Aβ sequence, such as the conversion of aspartate to isoaspartate at residue 23, have been reported to increase Aβ aggregation, potentially contributing to AD pathology [134]. Isoaspartate formation in the Aβ peptide sequence has been attributed to several factors, including oxidative stress, aging, and reduced activity of enzymes involved in protein repair mechanisms. The nonenzymatic conversion of aspartate residues to isoaspartate can lead to altered protein structure, stability, and function. In the case of Aβ peptides, isoaspartate formation promotes the formation of toxic oligomers and fibrils, which are central to the development of amyloid plaques in AD [135, 136].

Isoaspartate-modified Aβ peptides not only contribute to the formation of amyloid plaques but also have detrimental effects on neuronal function. They can impair synaptic plasticity, disrupt calcium homeostasis, induce neuroinflammation, and trigger oxidative stress, all of which are associated with neurodegeneration in AD. Additionally, isoaspartate-modified Aβ peptides have been shown to have a higher resistance to degradation and clearance mechanisms in the brain, leading to their prolonged accumulation [136, 137].

Interestingly, in hereditary cases and animal models of AD, there is typically an increase in the production of Aβ peptides or an elevated ratio of Aβ42 to Aβ40. This suggests that genetic mutations or experimental factors lead to an overproduction of Aβ, contributing to the development of AD pathology. However, in AD patients, the levels of Aβ in the CSF do not show an overall increase. Instead, there is a consistent observation of reduced levels of Aβ42 in the CSF, which serves as a biomarker for AD. This decrease in Aβ42 is likely due to impaired clearance mechanisms, resulting in the accumulation of Aβ in the brain, particularly in the form of amyloid plaques. The precise mechanisms underlying impaired clearance and the dynamics of Aβ metabolism in AD are still under investigation. Nonetheless, measuring Aβ42 levels in the CSF is an important diagnostic tool for AD, helping to assess disease progression and response to treatments [138].

### Aβ oligomers

In recent years, there has been mounting evidence indicating the significant role of oligomers in AD. Research experiments have demonstrated that oligomers possess toxic properties both in in vivo [139] and in vitro [140]. Furthermore, it has been observed that the learning and memory impairments caused by oligomers can be alleviated by promoting the formation of fibrils [141].

Earlier investigations utilizing the FAD APP Indiana mutation have revealed that the neurotoxic effects induced by Aβ do not necessarily rely on Aβ accumulation in plaques [142]. Animal models of AD have provided additional support for this notion, as the presence of oligomers in these models was associated with the manifestation of disease symptoms [143]. Moreover, the quantity of oligomers extracted from human AD brain tissue exhibited a stronger correlation with disease symptoms compared to the number of amyloid plaques [144, 145].

Aβ oligomers also exert detrimental effects on the brain, manifesting in synaptic dysfunction, excitotoxicity, and neuronal damage. By interfering with NMDA receptors, particularly NR2A [146] and GluN2B subunits [147], Aβ oligomers disrupt the delicate balance of calcium, resulting in an excessive influx of this ion into neurons. Consequently, synaptic plasticity and the ability to learn are impaired [148]. Moreover, Aβ oligomers disturb the equilibrium of glutamate, a primary excitatory neurotransmitter, and interact with synaptic proteins, leading to their depletion and compromising the release of neurotransmitters [20, 149]. The exact mechanisms underlying the inhibitory effects of Aβ oligomers on NMDA-mediated synaptic transmission are still not fully understood [150]. However, a study [151] sought to investigate this phenomenon by using brain extracts from AD patients and hippocampal slice cultures. The researchers focused on the impact of Aβ oligomers, specifically Aβ dimers, on NMDA receptor function. The study found that Aβ dimers, a specific type of Aβ oligomer, were particularly potent in inhibiting NMDA-mediated synaptic transmission. These dimers had a significant impact on the normal functioning of NMDA receptors, impairing synaptic transmission. Interestingly, higher molecular weight Aβ oligomers and insoluble aggregates were capable of releasing Aβ dimers, suggesting a dynamic relationship between different forms of Aβ in the brain. Additionally, Aβ oligomers disrupt the postsynaptic density, a specialized structure at the postsynaptic membrane, leading to the loss of dendritic spines and synapse loss.

In addition to synaptic dysfunction, Aβ oligomers also induce oxidative stress [152], which inflicts damage upon cellular components and impairs the defense mechanisms against oxidative damage. These oligomers disrupt the homeostasis of ions like calcium and the normal functioning of neurons, resulting in neuronal injury and, ultimately, cell death [153].

Notably, Aβ monomers undergo a structural change from their soluble form to a beta-sheet-rich conformation, leading to the formation of small soluble oligomers. These oligomers act as seeds and encourage the aggregation of Aβ peptides into larger insoluble aggregates, such as fibrils. The precise structures and sizes of Aβ oligomers are still under investigation, but it is believed that small soluble oligomers, such as dimers and trimers, are particularly neurotoxic [154, 155].

Impaired clearance mechanisms, which involve enzymatic degradation and cellular uptake by microglia [156] and other phagocytic cells, contribute to the accumulation of Aβ in the brain [156, 157]. However, Aβ oligomers can hinder the clearance of Aβ aggregates and can interact with microglia and disrupt their ability to effectively clear Aβ [158]. Aβ oligomers interfere with the internalization and degradation of Aβ by binding to microglial receptors, such as RAGE and low-density lipoprotein receptor-related protein 1 (LRP1). This impairs the uptake and clearance of Aβ aggregates by microglia, contributing to their accumulation in the brain [159, 160].

Aβ oligomers exert neurotoxic effects, contributing to neuronal damage and cell death. They interact with neuronal membranes, forming ion channels or pores that disrupt the ionic homeostasis of cells, particularly calcium dysregulation [161]. This excessive influx of calcium triggers downstream signaling pathways associated with oxidative stress, mitochondrial dysfunction, and synaptic impairment. Aβ oligomers also generate ROS, leading to oxidative damage to cellular components, including lipids, proteins, and DNA. The accumulation of oxidative damage further contributes to neuronal dysfunction and cell death [162, 163].

**Fig. 3:**
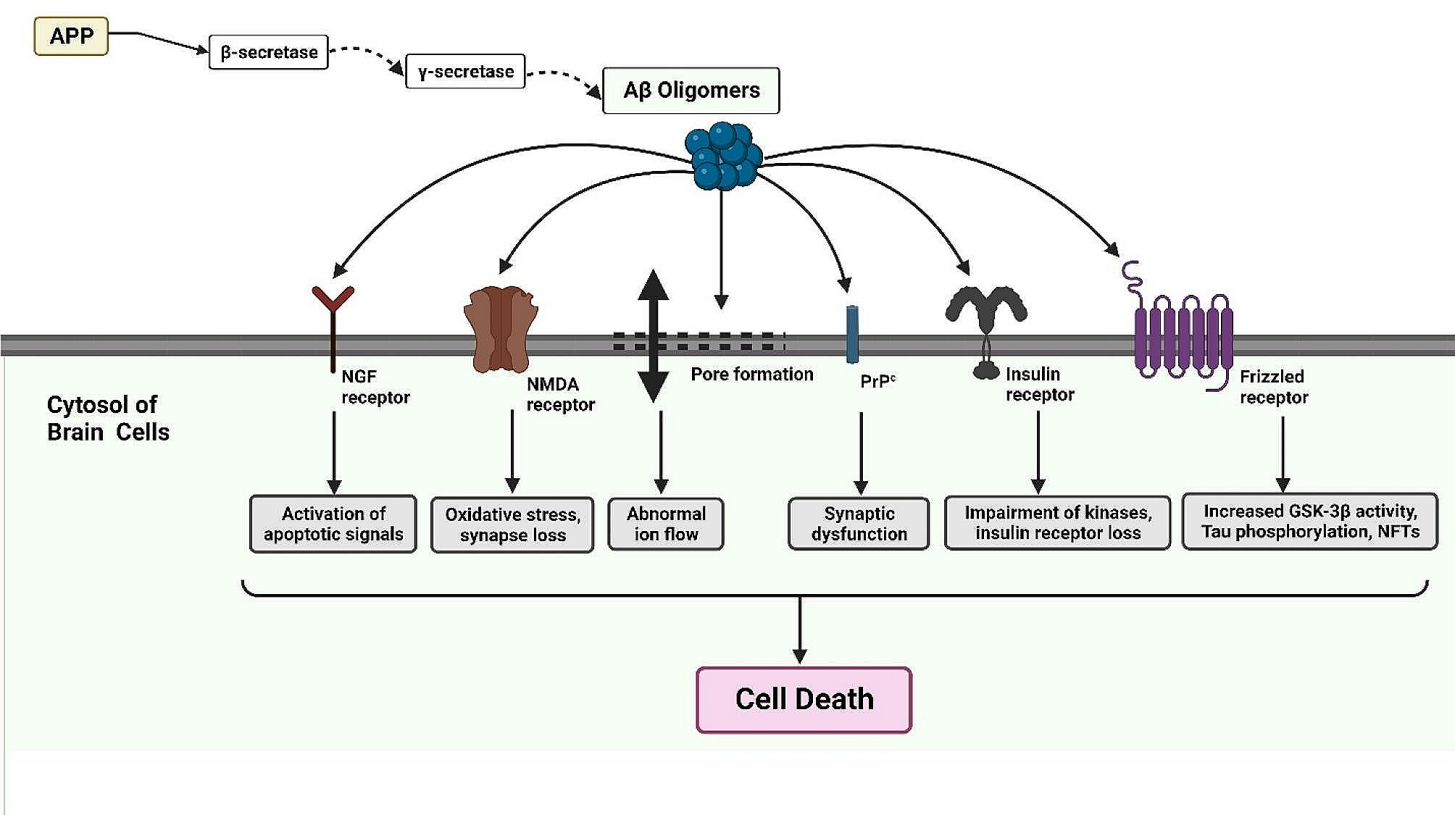
Aβ oligomers have a detrimental impact on various receptors in the brain, including Frizzled receptors, PrPc, NMDA receptors, insulin receptors, and NGF receptors. Their interaction with these receptors leads to tau phosphorylation, activation of GSK-3β, synaptic dysfunction, excitotoxicity, disruption of insulin signaling, impairment of NGF signaling, and ultimately, cell death. These complex interactions contribute to the progression of AD and underscore the importance of understanding and targeting Aβ oligomers to develop effective therapeutic strategies

Another mechanism by which Aβ oligomers contribute to Aβ accumulation is the impairment of proteostasis. Aβ oligomers disrupt the delicate balance of protein folding and degradation within neurons. They can interact with molecular chaperones, such as heat shock proteins (HSPs), and interfere with their function. Chaperones play a crucial role in facilitating proper protein folding and preventing protein aggregation. Aβ oligomers can sequester chaperones, leading to the misfolding and aggregation of Aβ and other proteins [164–167]. Furthermore, Aβ oligomers impair the activity of proteolytic systems, such as the ubiquitin-proteasome system and autophagy-lysosomal pathway. This hinders the degradation of misfolded proteins, including Aβ itself, contributing to their accumulation [168–171] (Fig. 3).

It is important to note that further research is needed to fully elucidate the underlying mechanisms and the precise role of Aβ oligomers in AD pathology. However, these findings provide valuable insights into the potential mechanisms by which Aβ oligomers contribute to neurodegeneration in AD.

### Aβ toxicity

Aβ toxicity is mediated by multiple mechanisms including oxidative stress, mitochondrial dysfunction, alterations in membrane permeability, inflammation, synaptic dysfunction, and excitotoxicity through interactions with neurotransmitter receptors [172–176].

The pro-oxidant effect of the Aβ peptide has been extensively studied using paramagnetic electron resonance (PER) techniques [177], although the exact mechanism behind this effect is still a subject of debate. Aβ possesses metal-binding sites within its first 15 amino acids, particularly with a high affinity for copper ions (Cu2+), and is known to interact with metallic chelants [56]. The binding of Aβ to Cu2 + occurs through the nitrogen atoms in histidine residues’ imidazole rings, with oxygen atoms provided by tyrosine 10, glutamic acid 5 (Glu5), or water molecules [178]. These interactions play a role in the pro-oxidant properties of Aβ, contributing to oxidative damage.

The Aβ peptide has been observed to possess the ability to reduce Cu2 + and Fe3 + ions to their lower oxidation states, namely Cu + and Fe2 + respectively. Consequently, molecular oxygen can react with these reduced metals, resulting in the generation of superoxide anions. These anions can then combine with hydrogen atoms to form hydrogen peroxide. Furthermore, hydrogen peroxide may subsequently react with additional reduced metal ions, ultimately leading to the production of hydroxyl radicals through a process known as the Fenton reaction. Additionally, the radical form of Aβ has the capacity to extract protons from neighboring lipids or proteins. This extraction can lead to the formation of lipid peroxides and carbonyls [178]. Notably, studies have provided evidence supporting the role of metals in Aβ’s toxicity. In these experiments, the removal of metals from the reaction medium or the use of deferoxamine, a metal chelator, effectively reduced the toxicity levels of Aβ in cellular cultures. This suggests that the presence of metals, and their interaction with Aβ, contribute to the harmful effects of Aβ in cellular systems [179, 180].

It has been proposed that the reduction of metals is facilitated by a methionine residue located at position 35, as its sulfide group readily donates electrons [181]. Supporting this hypothesis, copper-bound methionine sulfoxide has been found within the amyloid plaques of AD patients [182]. However, the exact role of this residue remains a topic of discussion, as one study demonstrated the oxidation of neurotransmitters even when Aβ peptides lacking the Met35 residue were bound to metals. Additionally, external reducers such as dopamine or ascorbate have been suggested to initiate redox cycles of metallic ions without requiring peptide autoxidation [183]. Furthermore, the formation of tyrosyl radicals from the 10th tyrosine residue of Aβ contributes to the cross-linking of Aβ molecules, leading to the formation of Aβ oligomers [178, 184].

An additional mechanism related to Aβ-induced toxicity involves the upregulation of the divalent metal transporter 1 (DMT1). Increased expression of DMT1 has been observed in senile plaques of AD patients, as well as in APP/SS1 transgenic mice and cellular lines that overexpress APP. This upregulation of DMT1 is associated with higher levels of iron in cells exposed to Aβ. These findings suggest that disturbances in iron homeostasis may contribute to the increased oxidative stress induced by Aβ. The dysregulation of iron levels and the subsequent generation of reactive oxygen species can further exacerbate the pathological processes associated with AD [185].

The severity of synaptic loss in AD patients has been shown to have a stronger correlation with cognitive impairment rather than the accumulation of Aβ deposits or neurofibrillary tangles [186]. Notably, studies have consistently reported significant reductions in cortical synapses, both in terms of overall numbers and per neuron, in AD patients. Furthermore, there is a notable decrease in the levels of presynaptic markers (such as synaptophysin) and postsynaptic markers (such as synaptopodin and PSD-95) in AD patients compared to healthy individuals [187]. Interestingly, disturbances in synaptic transmission have been observed early in the disease progression, occurring prior to the development of typical neuropathological lesions [188]. Aβ soluble oligomers, rather than fibrillar forms, have been identified as culprits in impairing LTP through various mechanisms, including the reduction of PSD-95 levels and negative regulation of glutamatergic receptors [189]. However, it is important to note that fibrillar forms of Aβ also contribute to synaptic damage in AD. Specifically, Aβ aggregates have been found to inhibit NMDAr-dependent LTP and promote long-term depression (LTD) in hippocampal neurons, potentially linked to disruptions in glutamate reuptake [190]. Some experiments have provided insights into the damaging effects of Aβ oligomers on synaptic transmission, although their precise mechanism is still unclear. Notably, when Aβ 1–42 was administered intra-axonally in a giant squid’s axon, it resulted in alterations in electrophysiological parameters and bidirectional fast axonal transport. However, no observable effect was observed when Aβ oligomers were administered at the extracellular level [191, 192].

In contrast, an intriguing experimental report has provided evidence that synaptic activity exerts a dual effect on Aβ. Firstly, it reduces the intracellular levels of Aβ, potentially mediated by the action of neprilysin. Secondly, synaptic activity promotes the extracellular secretion of Aβ, leading to a decrease in its synaptic toxicity. These findings strongly support the hypothesis that the primary mechanism of Aβ’s toxicity occurs within the intracellular milieu. Furthermore, recent studies have unveiled the crucial role of Tau protein in mediating the detrimental effects of Aβ on synaptic functionality. Notably, investigations have demonstrated that hippocampal slices obtained from animals lacking Tau protein exhibit remarkable resistance to the harmful impact of Aβ 1–42 on LTP [193].

### Aβ accumulation and BBB

Aβ accumulation is indeed associated with the compromise of the BBB in certain conditions, particularly in AD [194]. For instance, Aβ plaques has been shown to disrupt the BBB, as it induces inflammation and oxidative stress, and it also directly interacts with BBB components; as a result, the BBB becomes compromised, which leads to heightened permeability and the entry of detrimental substances into the brain. Inflammatory mediators, in such condition, contribute to the damage of endothelial cells, thereby disrupting tight junctions (TJs) and persisting BBB permeability [195, 196]. A study provided evidence that different concentrations of beta-amyloid (Aβ1–42), both high and low, can cause changes in TJ proteins, specifically claudin-5, occludin, and zona occludens-1 (ZO-1). These alterations in TJ proteins resulted in increased permeability of the BBB, as demonstrated by an FD-40 penetration assay [197]. This suggests that Aβ has the ability to disrupt the distribution of TJ proteins, leading to compromised integrity of the BBB [198].

### Aβ and prion protein (PrP)

An intriguing recent discovery involves the interaction between Aβ and cellular PrP. A study highlighted in the statement revealed that Aβ oligomers, consisting of approximately 100 molecules, exerted their inhibitory effect on NMDA-mediated synaptic transmission only when they could bind to the cellular form of PrP. In mice lacking PrP, this interaction was absent, and Aβ peptides did not exhibit inhibitory or toxic effects. Moreover, other interactions between APP or Aβ with PrP have been described, along with reciprocal modulation of AD or scrapie disease progression in mice. These findings suggest that the interaction between Aβ and PrP plays a significant role in the pathogenesis and progression of AD and related prion diseases. [199]. Notably, cellular PrP has been shown to inhibit BACE1-mediated Aβ production. By inhibiting BACE1, PrP effectively reduces the production of Aβ. This finding suggests that PrP may play a role in regulating Aβ levels and could potentially have implications for the development of therapeutic strategies targeted at reducing Aβ production in AD [200].

### Aβ and glial cells

Research on the relationship between Aβ and glial cells is expanding to understand if neuroinflammation triggers or sustains Aβ dyshomeostasis; thus far, most studies in vitro and in murine models have supported neuroinflammation as a key pathogenic event in AD. In the context of AD, there is interaction between different Aβ species and receptors found on microglia and astrocytes, which initiates an innate immune response. The accumulation of Aβ in the brain triggers a process known as microglial “priming,” making them more susceptible to secondary inflammation [201, 202]. Consequently, activated microglia become a characteristic pathological feature of AD. These activated microglia surround Aβ plaques and fibrils, forming a protective barrier and contributing to the clearance of Aβ from the brain [203–205]. However, when microglial activity becomes dysregulated, it can worsen the aggregation of brain proteins, further exacerbating the progression of the AD [205, 206].

The presence of Aβ aggregates, including oligomers, protofibrils, and fibrils, promotes inflammation [207–209]. Microglia express cell surface receptors that enable them to bind to these aggregates, leading to neuroinflammation and neurodegeneration. Neurotrophic factor TGF-β1 plays a crucial role in stimulating Aβ clearance by microglia [210, 211]. On the other hand, TNF-α plays a pro-inflammatory role in AD [208, 211–214]. To clarify, TGF-β1 promotes the clearance of Aβ by microglia, enhancing their phagocytic activity and potentially reducing Aβ accumulation in the brain. This mechanism may exert a protective effect against the pathological features of AD [215, 216]. Conversely, TNF-α, a pro-inflammatory cytokine, contributes to the immune response and inflammation in AD. Elevated levels of TNF-α in AD brains indicate the presence of a pro-inflammatory environment, leading to chronic inflammation and neuronal damage. The immune response and inflammation in AD involve complex interactions among various factors and cell types [217, 218]. Further investigation is necessary to fully comprehend these processes and explore their potential as therapeutic targets for AD treatment.

During early AD pathogenesis, Aβ oligomers, protofibrils, and fibrils accumulate in the extracellular space, triggering a pathological cascade [219]. Microglia are responsible for phagocytosing these Aβ forms and clearing dying cells. The function of microglia, the immune cells of the brain, is modulated by TREM2 (Triggering Receptor Expressed on Myeloid Cells 2). TREM2 plays a role in the response to Aβ plaques, which are characteristic features of AD. When microglia detect Aβ plaques, TREM2 activation stimulates the production of inflammatory cytokines. Inflammatory cytokines are signaling molecules that can promote an immune response and contribute to inflammation. In the context of AD, the activation of microglia and the release of inflammatory cytokines, facilitated by TREM2, are part of the immune response against Aβ plaques. However, excessive or chronic inflammation can have detrimental effects on neuronal health. Therefore, the regulation of microglial function by TREM2 and the balance of inflammatory responses are important areas of study in understanding the underlying mechanisms of AD pathology [210, 220–222]. In addition to microglia, hypertrophic reactive astrocytes can also surround Aβ plaques. Upon exposure to Aβ, these astrocytes release pro-inflammatory molecules, contributing to the inflammatory environment in the brain [213, 223–225].

Astrocytes are integral to the brain’s response to Aβ accumulation in AD, influencing Aβ dynamics through various cellular mechanisms. These glial cells can both promote and mitigate amyloid pathology, playing a multifaceted role in disease progression [226–228].

Astrocytes contribute to amyloid accumulation primarily through impaired Aβ clearance. They are equipped with enzymes such as neprilysin and IDE that degrade Aβ. However, in AD, the expression and activity of these enzymes are often reduced, leading to less efficient degradation of Aβ and its subsequent accumulation in the extracellular space [229, 230]. Additionally, astrocytes usually uptake Aβ via receptors like LRP1, but this process becomes less effective in AD, further contributing to the build-up of Aβ [231, 232].

Inflammatory responses also play a significant role in promoting amyloid accumulation. Reactive astrocytes, which are characterized by hypertrophy and increased expression of glial fibrillary acidic protein (GFAP) [227], release pro-inflammatory cytokines such as IL-1β, TNF-α, and IL-6. These cytokines sustain a chronic inflammatory environment that disrupts normal Aβ processing and clearance [233–235]. Reactive astrocytes also produce ROS, which cause oxidative stress and damage neuronal and glial cells, further impairing Aβ metabolism [236, 237]. Furthermore, astrocytes support amyloid plaque formation through gliosis and scar formation. Indeed, astrocytes surrounding Aβ plaques undergo gliosis, forming a glial scar that isolates these plaques. While this may protect surrounding neurons from the toxic effects of Aβ, it also creates a barrier that prevents efficient clearance of plaques [238, 239]. Additionally, astrocytes secrete ApoE and other molecules that facilitate Aβ aggregation, stabilizing the plaques and potentially exacerbating amyloid pathology [240].

The interaction between astrocytes and microglia is pivotal in the context of Aβ accumulation and neuroinflammation in AD. These interactions, mediated through complex signaling pathways, significantly impact disease progression. Astrocytes and microglia communicate extensively via cytokines and chemokines, modulating each other’s activity [241]. Astrocytes release chemokines like CCL2 (MCP-1), which attract microglia to sites of Aβ deposition. Once there, astrocyte-derived cytokines such as IL-1β and TNF-α can activate microglia, inducing a reactive state. Activated microglia, in turn, release their own cytokines, creating a feedback loop that amplifies neuroinflammation. This bidirectional signaling can sustain a chronic inflammatory state that hinders Aβ clearance and promotes further amyloid deposition [242–244].

#### Aβ and nuclear factor-κB (NF-κB)

The NF-κB family, comprising NF-κB1 (p105/p50), NF-κB2 (p100/p52), RelA (p65), RelB, and c-Rel, is pivotal in cellular processes, particularly inflammatory responses. NF-κB governs a multitude of genes, many of which are implicated in inflammation. Its activation triggers the transcription of target genes, thereby fostering an inflammatory response. NF-κB activation occurs via two primary pathways: The canonical pathway and the non-canonical pathway. The canonical pathway orchestrates inflammatory responses by sequestering NF-κB in the cytoplasm and subsequently liberating dimers [245]. Conversely, the non-canonical pathway is initiated by TNFR superfamily members, leading to the recruitment and activation of NF-κB-inducing kinase (NIK). Dysregulation of NF-κB signaling is associated with diseases such as chronic inflammation and cancer [246].

In AD, Toll-like receptors (TLRs) are overexpressed on microglia and neurons, resulting in the activation of the NF-κB signaling pathway and subsequent production of proinflammatory factors [247]. Early activation of microglia plays a pivotal role in AD development, contributing to the establishment of chronic inflammation. Therefore, gaining a comprehensive understanding of NF-κB’s involvement in AD is essential. Currently, there is ongoing research and development of drugs, including NF-κB inhibitors, aimed at targeting this pathway in the context of AD [248].

**Fig. 4:**
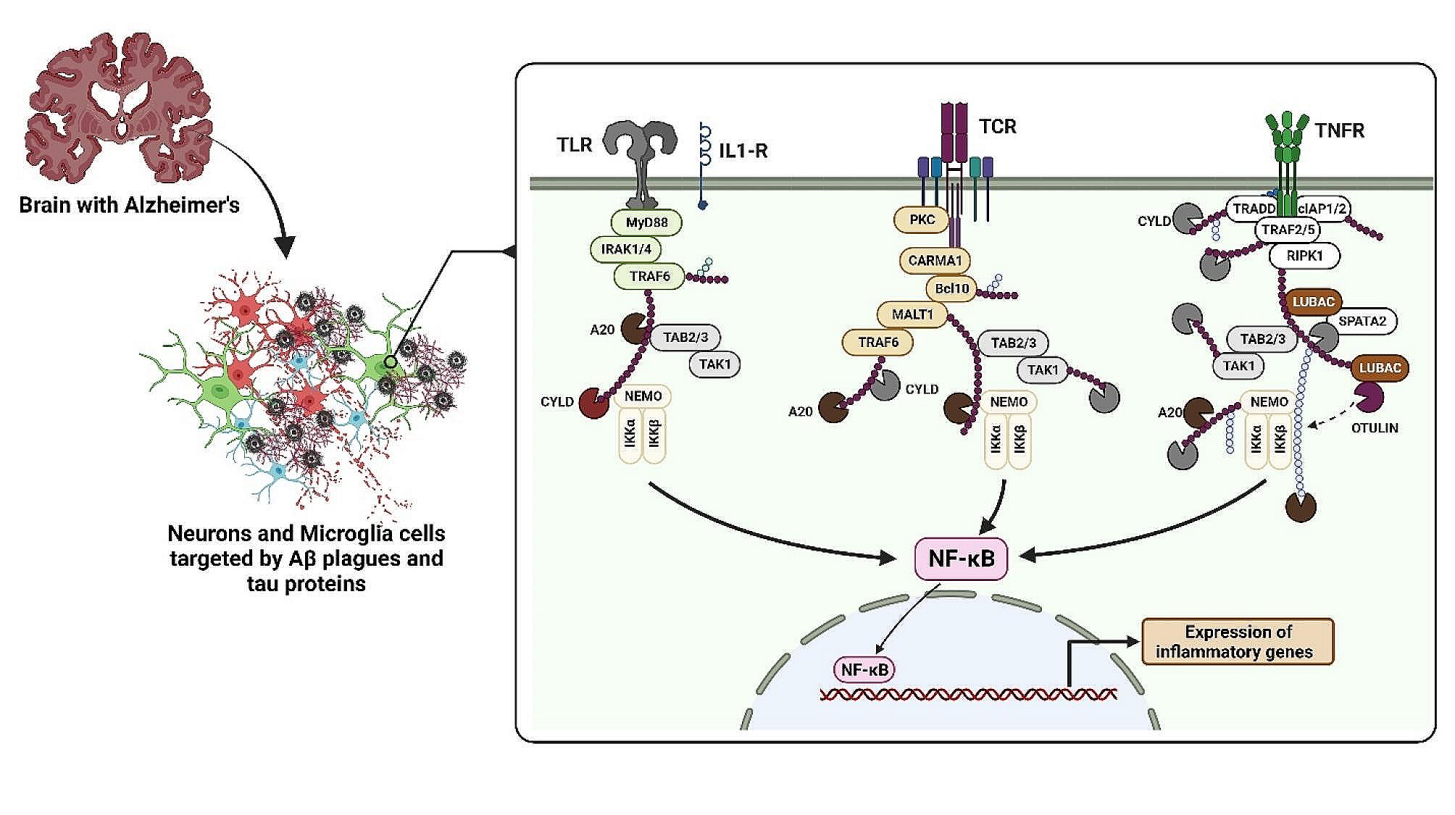
Aβ plays a role in triggering the activation of NF-κB, a central regulator of inflammation, through various pathways in neurons and microglia cells, contributing to the development of AD. In microglia cells, one pathway by which Aβ induces NF-κB activation is the Toll-like receptor (TLR) pathway. Aβ interacts with TLR2 and TLR4, leading to the recruitment of adaptor proteins like MyD88. This activates downstream signaling molecules, including interleukin-1 receptor-associated kinases (IRAKs). The phosphorylation of IRAKs leads to the activation of the transforming growth factor-beta-activated kinase 1 (TAK1) complex. The TAK1 complex, along with the inhibitor of κB kinase (IKK) complex, phosphorylates and degrades IκB, releasing NF-κB from its inhibitory state. NF-κB then translocates into the nucleus, where it forms a transcriptional complex with coactivators and binds to κB sites in the promoters of proinflammatory genes such as IL-1β and TNF-α, promoting their expression. In neurons, Aβ can activate NF-κB through the T-cell receptor (TCR) pathway. Aβ peptides interact with major histocompatibility complex class II (MHC-II) molecules on antigen-presenting cells like microglia. This triggers TCR signaling in T cells, leading to the release of proinflammatory cytokines, including IFN-γ. IFN-γ binds to its receptors on neurons, initiating Janus kinase (JAK) and signal transducer and activator of transcription (STAT) signaling. The JAK-STAT pathway activates transcription factors, including STAT1 and STAT3, which collaborate with NF-κB to enhance its activity. This collaboration promotes the expression of proinflammatory genes. Furthermore, Aβ can activate NF-κB through the tumor necrosis factor receptor (TNFR) pathway in both neurons and microglia cells. By interacting with TNFR, Aβ triggers the recruitment and activation of TNFR-associated factor (TRAF) proteins, particularly TRAF2 and TRAF6. These proteins activate the IKK complex, which includes IKKα, IKKβ, and IKKγ. The activated IKK complex phosphorylates IκB, leading to its ubiquitination and degradation. The degradation of IκB releases NF-κB, allowing its translocation into the nucleus. In the nucleus, NF-κB forms a transcriptional complex that promotes the transcription of proinflammatory genes. Overall, these pathways highlight how Aβ can initiate NF-κB activation in both microglia cells and neurons, leading to the expression of proinflammatory genes and contributing to the inflammatory processes observed in AD [252, 253]

In addition, the activation of NF-κB by *bacteroides fragilis* lipopolysaccharide triggers a cascade of events, leading to increased Aβ plaque accumulation and tau hyperphosphorylation. Consequently, this results in the impairment of oligodendrocytes, causing myelin injury and neurotoxicity [249]. Moreover, NF-κB activation in astrocytes fosters Aβ42 accumulation and the production of pro-inflammatory cytokines, such as IL-1, IL-6, and TNF-α, thereby intensifying neurodegeneration in AD [250]. The intricate involvement of NF-κB signaling in reactive microglia and astrocytes underscores its profound impact on AD progression, highlighting its potential as a promising therapeutic target [251]. (For further information, see [252, 253]) (Fig. 4).

In AD, elevated levels of NF-κB have been also observed in the cerebral cortex, coinciding with increased levels of BACE1. NF-κB, particularly the p65 subunit, binds to the BACE1 promoter, leading to the upregulation of β-secretase expression and the amyloidogenic processing of APP [254]. This process contributes to the formation of amyloid plaques. Additionally, Aβ peptides can stimulate NF-κB activation, further exacerbating AD pathology [255]. Notably, Aβ40 peptide activates NF-κB and induces the expression of pro-apoptotic genes, while also promoting the accumulation of Aβ42 aggregates [256]. Aβ (25–35) peptide causes neuronal toxicity through oxidative stress and is accompanied by increased NF-κB signaling [257]. Understanding the role of NF-κB in AD is crucial for developing potential therapeutic interventions, including NF-κB inhibitors.

### Aβ and tau protein

In AD, the accumulation of Aβ plaques and the formation of NFTs composed of abnormal tau protein are two key pathological features. Aβ accumulation is believed to initiate a cascade of events that lead to tau pathology [258]. Aβ can promote the hyperphosphorylation of tau, disrupt the stability of microtubules, induce oxidative stress and inflammation, and impair synaptic function [259].

Indeed, studies have demonstrated that incubating neurons with a concentration of 5 µM Aβ can activate the p38 MAPK signaling pathway, resulting in the hyperphosphorylation of tau protein [260–262]. This activation of p38 MAPK disrupts the normal function of tau, leading to the formation of NFTs and instability of microtubules. The specific mechanisms by which Aβ activates the p38 MAPK pathway and induces tau hyperphosphorylation are still being investigated. It is believed that Aβ can trigger intracellular signaling events, potentially involving receptors or oxidative stress, which culminate in the activation of p38 MAPK. Once activated, p38 MAPK can directly phosphorylate tau or activate downstream kinases that contribute to tau hyperphosphorylation. This abnormal phosphorylation of tau impairs its ability to bind to microtubules, leading to their destabilization and subsequent disruption of neuronal structure and function [41, 263].

These processes contribute to the aggregation of tau into NFTs, which further disrupt neuronal function and contribute to cognitive decline. Additionally, tau pathology can spread throughout the brain, propagating the disease process from one region to another [264]. The interaction between Aβ and tau appears to have synergistic effects, exacerbating neuronal dysfunction and neurodegeneration in AD [265]. Understanding the relationship between these two pathological features is crucial for developing effective treatments for AD.

### Aβ and APOE ε4 allele

Most importantly, the presence of the APOE ε4 allele, a genetic variant associated with AD, is a significant risk factor for both late-onset and early-onset forms of the disease [266]. Individuals carrying the APOE ε4 allele may experience earlier cognitive decline, even before the age of 60, compared to non-carriers [267]. Of note, homozygosity for the APOE ε4 allele further increases the risk of developing AD [268]. APOE ε4 influences various brain signaling pathways involved in lipid transport, synaptic function, glucose metabolism, and cerebrovascular health [269]. The APOE ε4 allele is associated with increased accumulation of Aβ plaques, neurotoxic Aβ species, and intraneuronal Aβ accumulation. It also correlates with higher cerebral Aβ deposition as detected by neuroimaging and cerebrospinal fluid biomarkers. The impact of APOE ε4 on AD risk and progression is likely mediated through its effects on Aβ metabolism. Age-related changes and interactions between APOE ε4 and metabolic processes further exacerbate Aβ-related pathology [270]. Understanding the role of APOE ε4 and its interaction with age and Aβ accumulation is important for developing predictive models and potential therapeutic strategies for AD.

The APOE genotype has a profound impact on Aβ deposition in both humans and animal models. Specifically, individuals with an APOE ε4 allele exhibit a strong association with increased levels of Aβ, including the toxic oligomeric form detected in post-mortem AD brains [271, 272]. Moreover, throughout the progression of the disease, APOE ε4 exacerbates intra-neuronal Aβ deposition [273], plaque formation [274, 275], and the development of cerebral amyloid angiopathy within the cerebrovasculature [276, 277]. Brain Aβ metabolism is differentially influenced by ApoE isoforms [278], and when combined with amyloid mouse models, the presence of apoE4 intensifies the severity of Aβ deposition compared to apoE2 or apoE3 [279–281].

One primary mechanism is impaired Aβ clearance, as APOE ε4 is less efficient in lipid transport and has reduced affinity for receptors like LRP1 and SORL1, leading to decreased Aβ removal from the brain. APOE ε4 also promotes Aβ aggregation by enhancing fibril formation and influences APP processing, increasing the production of the aggregation-prone Aβ42 isoform [282, 283].

Neuroinflammation is another critical pathway, with APOE ε4 associated with increased activation of microglia and astrocytes, leading to the release of pro-inflammatory cytokines and ROS, contributing to neurotoxicity and further Aβ accumulation [284, 285]. Additionally, APOE ε4 is linked to BBB dysfunction, allowing more peripheral Aβ and inflammatory factors to enter the brain [286, 287]. Mitochondrial dysfunction and oxidative stress are further exacerbated by APOE ε4, leading to reduced ATP production and increased ROS, which damage cellular components. This isoform also disrupts synaptic function by promoting the accumulation of toxic Aβ oligomers, leading to cognitive decline [288, 289].

### Aβ and BACE1

In mouse models, the absence of the BACE1 protein, known as β-secretase, completely eliminates β-secretase activity in the brain and cultured neurons [290, 291]. In contrast, mice overexpressing a mutated form of the APP gene associated with AD produce high levels of brain Aβ and develop Aβ plaques. By breeding BACE1-deficient mice with the APP-overexpressing mice, it was found that the resulting mice lacking BACE1 lacked all forms of brain Aβ, APPsβ, and C99, proving that BACE1 is the primary β-secretase required for Aβ generation in the brain [291, 292]. These findings highlight the crucial role of BACE1 in the production of Aβ and provide insights into the mechanisms underlying AD.

Recent studies have shown that BACE1 deficiency and the ablation of Aβ can rescue memory deficits in Tg2576 mice, a type of AD brain [293]. The study revealed that BACE1-/-•Tg2576 bigenic mice, which lack Aβ, did not exhibit memory deficits or cholinergic dysfunction in the hippocampus. In contrast, Aβ-overproducing Tg2576 monogenic mice displayed pronounced deficits in memory function [294]. These findings strongly support BACE1 as a promising therapeutic target for AD and provide direct evidence for the amyloid hypothesis in living organisms. In aged APP/PS1 double transgenic mice, which show accelerated Aβ accumulation and memory deficits associated with AD, the deletion of BACE1 completely eliminates the deposition of amyloid plaques and prevents deficits in spatial reference memory [295–298]. Research using BACE1 knockout mice has shown impairments in emotional and cognitive processes, which may indicate mechanism-based toxicities from total BACE1 inhibition [293, 294, 297].

BACE1 is a critical enzyme for the generation of Aβ, implying that Aβ may have regular physiological functions related to memory, neuronal function, and potentially potassium channel expression regulation [293]. Disrupting Aβ production is associated with impaired memory performance [299, 300]. However, BACE1 deficiency does not uniformly affect all types of learning associated with the hippocampus, indicating that Aβ’s role in cognitive function and its normal physiological function in vivo require further research [293].

Developing BACE1 inhibitors to completely suppress its enzymatic activity in vivo may pose challenges. However, a study by Singer et al. demonstrated that partial reduction of BACE1 through RNA interference improved amyloid pathology and cognitive deficits in APP Tg mice, suggesting that even moderate inhibition of BACE1 could be therapeutically beneficial. These findings shed light on the potential of targeting BACE1 activity as a treatment strategy for AD, emphasizing the importance of exploring approaches that modulate BACE1 levels or activity rather than complete inhibition [301]. Moreover, BACE1 knockout mice exhibit normal spatial memory function, suggesting that complete inhibition of BACE1 may not impact learning abilities. However, the dosage of BACE1 affects the burden of Aβ plaques, particularly in young animals, indicating that BACE1 plays a role in Aβ production. These findings emphasize the complex relationship between BACE1, Aβ burden, and cognitive function, highlighting the need for further research to understand the precise role of BACE1 in AD [294].

Nonetheless, a study revealed that in older mice, decreasing BACE1 levels did not affect the burden of Aβ plaques, indicating that BACE1 is not a limiting factor in aged mice. Additionally, older mice with a 50% reduction in BACE1 levels, specifically in the APPswe; PS1ΔE9 model, showed significant impairment in the Morris water maze, indicating that partial BACE1 suppression alone is insufficient to improve cognitive deficits in aged mice. The age-dependent effects of partially suppressing BACE1 expression seem to be complex, potentially influenced by different forms of the APP [294, 302].

### Aβ and receptors

Soluble oligomeric forms of Aβ have been found to interact with a range of receptors, including lipids, proteoglycans, and specific proteins present on the surface of neuronal cells. Several receptors associated with Aβ toxicity have been identified, such as the Aβ-binding p75 neurotrophin receptor (P75NRT), the LRP, cellular PrPc, metabotropic glutamate receptors (mGluR5), α subunit containing nicotinic acetylcholine receptor (α7nAChR), NMDAR, β-adrenergic receptor (β-AR), erythropoietin-producing hepatoma cell line receptor (EphR), and paired immunoglobulin-like receptor B (PirB). These receptors play a role in mediating the toxic effects of Aβ and contribute to the pathogenesis of AD [303].

The interactions between Aβ and these receptors are believed to generate and transmit neurotoxic signals within neurons, leading to cellular defects such as mitochondrial dysfunction and activation of the endoplasmic reticulum stress response. These cellular defects contribute to the progressive neurodegeneration observed in AD. Furthermore, some of these Aβ receptors are likely to internalize Aβ peptides into neurons, leading to the manifestation of distinct cellular defects. This internalization process may contribute to the spread and propagation of Aβ pathology within the brain [304, 305].

On the whole, the extracellular accumulation of Aβ in neuritic plaques and its binding to various receptors are key features of AD. The interaction between Aβ and these receptors can trigger neurotoxic signals, resulting in cellular defects and contributing to the progression of the disease. Understanding these Aβ/receptor interactions is important for unraveling the underlying mechanisms of AD’s pathology and developing potential therapeutic interventions. Table 1 shows summarize receptors affected by Aβ in AD.

**Table 1:** List the receptors in AD that are impacted by Aβ

Receptor	Function	Results in AD	Ref
Aβ-binding p75 neurotrophin receptor (P75NTR)	In AD, Aβ activates P75NTR, triggering signaling pathways promoting neuronal death.	Contributes to progressive neurodegeneration in AD.	[306–308]
Low-density lipoprotein receptor-related protein (LRP)	Its dysfunction leads to Aβ accumulation.	Impaired Aβ clearance contributes to plaque formation in AD.	[309, 310]
Cellular prion protein (PrPc)	Interaction with Aβ in AD contributes to neurotoxic effects and possible aggregation.	Role in Aβ-induced cellular events and possible aggregation.	[150, 311]
Metabotropic glutamate receptor 5 (mGluR5)	Aβ-mGluR5 interaction disrupts synaptic function, contributing to cognitive deficits.	Disruption leads to synaptic dysfunction and cognitive deficits.	[150, 312, 313]
α7 subunit-containing nicotinic acetylcholine receptor (α7nAChR)	Aβ-α7nAChR interaction disrupts cholinergic signaling, contributing to cognitive deficits.	Disruption impairs cholinergic signaling and cognitive functions.	[314]
N-methyl-D-aspartic acid receptor (NMDAR)	Aβ-NMDAR interaction disrupts function, leading to impaired plasticity and synaptic loss.	Dysfunction contributes to synaptic loss and cognitive decline.	[315, 316]
β-adrenergic receptor (β-AR)	Aβ-β-AR interaction triggers neurotoxic signals within neurons.	Contributes to cellular dysfunction and neuronal signaling issues.	[155, 317]
Erythropoietin-producing hepatoma cell line receptor (EphR)	Aβ-EphR interaction disrupts synaptic function and connectivity.	Leads to impaired synaptic function and neuronal connectivity.	[318–320]
Paired immunoglobulin-like receptor B (PirB)	Aβ-PirB interaction disrupts normal function, impacting synaptic plasticity and connectivity.	Results in disrupted synaptic plasticity and neuronal connectivity.	[321]

## Prospective and conclusion

AD poses a significant challenge in the field of neuroscience, primarily due to the complex nature of its progression, which involves various interconnected factors. A key aspect of understanding AD lies in comprehending how Aβ is metabolized and cleared in the brain. Researchers are extensively exploring the molecular mechanisms governing Aβ production, aggregation, and clearance in order to gain crucial insights into the underlying processes. Advanced imaging techniques, proteomics, and genetic studies are indispensable tools that provide deeper insights into Aβ accumulation and pave the way for the identification of novel therapeutic targets and strategies aimed at modulating its metabolism and enhancing its clearance.

The pursuit of potential treatments for AD revolves around innovative approaches targeting Aβ. Promising avenues include monoclonal antibodies, small molecules, and gene therapies, which aim to either reduce Aβ production or enhance its clearance from the brain. These strategies hold immense potential in combatting Aβ accumulation and its detrimental effects, offering the possibility of altering the course of the disease. Furthermore, researchers acknowledge the complexity of AD and the need to address multiple pathological processes simultaneously. Combination therapies that target various aspects of AD pathology, beyond Aβ alone, represent a compelling approach. By concurrently addressing neuroinflammation, abnormalities in tau proteins, synaptic dysfunction, and Aβ, these combination therapies offer a more comprehensive approach to mitigating AD progression. In addition, longitudinal studies that observe individuals over extended periods are pivotal in shaping our understanding of AD progression and treatment responses. These studies provide invaluable insights into the dynamic nature of the disease, informing the refinement of treatment strategies and enhancing the effectiveness of personalized medicine approaches.

In the realm of AD studies, it is crucial to acknowledge that Aβ plays a dual role and that comprehensive investigations must encompass other aspects related to Aβ as well as the broader factors contributing to AD development. While Aβ is strongly associated with AD and its accumulation is a characteristic feature, its precise role and impact on disease progression remain complex and multifaceted. AD is a multifactorial disease influenced by neuroinflammation, tau pathology, synaptic dysfunction, vascular factors, genetic predispositions, and lifestyle factors, among others. Understanding the intricate interplay between Aβ and these various factors is vital for a holistic understanding of AD pathogenesis and for identifying novel therapeutic targets and strategies.

## Data Availability

All data is within the paper.

## Abbreviations

AD: Alzheimer’s disease
Aβ: amyloid-beta
NFTs: neurofibrillary tangles
APP: amyloid precursor protein
sAPPα: soluble α-APP fragments
AICD: amyloid precursor protein intracellular domain
TACE: tumor necrosis factor-converting enzyme
BACE: beta-Site APP Cleaving enzyme
NSCs: neural stem cells
PI-3K: phosphatidylinositol 3-kinase
LTP: long-term potentiation
BBB: blood brain barrier
IDE: insulin-degrading enzyme
PER: paramagnetic electron resonance
DMT1: divalent metal transporter 1
LTD: long-term depression
PrP: prion protein
TREM2: triggering receptor expressed on myeloid cells 2
NF-κB: nuclear factor-κB
TLRs: Toll-like receptors

## References

[1] Castellani RJ, Rolston RK, Smith MA (2010). Alzheimer disease. Dis Mon.

[2] Sosa-Ortiz AL, Acosta-Castillo I, Prince MJ (2012). Epidemiology of dementias and Alzheimer’s Disease. Arch Med Res.

[3] Cullum CM, Rosenberg RN (1998). Memory loss—when is it Alzheimer Disease?. JAMA.

[4] Bloom GS (2014). Amyloid-β and tau: the trigger and bullet in Alzheimer disease pathogenesis. JAMA Neurol.

[5] Chen ZR, Huang JB, Yang SL, Hong FF. Role of Cholinergic Signaling in Alzheimer’s Disease. Molecules. 2022;27(6). 10.3390/molecules27061816. 10.3390/molecules27061816 PMC8949236 35335180

[6] Nordengen K, Kirsebom B-E, Henjum K, Selnes P, Gísladóttir B, Wettergreen M (2019). Glial activation and inflammation along the Alzheimer’s disease continuum. J Neuroinflamm.

[7] Bhatia S, Rawal R, Sharma P, Singh T, Singh M, Singh V (2022). Mitochondrial dysfunction in Alzheimer’s Disease: opportunities for Drug Development. Curr Neuropharmacol.

[8] Govindpani K, McNamara LG, Smith NR, Vinnakota C, Waldvogel HJ, Faull RL, Kwakowsky A. Vascular Dysfunction in Alzheimer’s Disease: A Prelude to the Pathological Process or a Consequence of It? J Clin Med. 2019;8(5). 10.3390/jcm8050651. 10.3390/jcm8050651 PMC6571853 31083442

[9] Ge M, Zhang J, Chen S, Huang Y, Chen W, He L, Zhang Y (2022). Role of Calcium Homeostasis in Alzheimer’s Disease. Neuropsychiatr Dis Treat.

[10] Cassidy L, Fernandez F, Johnson JB, Naiker M, Owoola AG, Broszczak DA (2020). Oxidative stress in alzheimer’s disease: a review on emergent natural polyphenolic therapeutics. Complement Ther Med.

[11] Pelucchi S, Gardoni F, Di Luca M, Marcello E (2022). Synaptic dysfunction in early phases of Alzheimer’s Disease. Handb Clin Neurol.

[12] Mohandas E, Rajmohan V, Raghunath B (2009). Neurobiology of Alzheimer’s disease. Indian J Psychiatry.

[13] Azargoonjahromi A, Abutalebian F (2024). Unraveling the therapeutic efficacy of resveratrol in Alzheimer’s disease: an umbrella review of systematic evidence. Nutr Metabolism.

[14] Prvulovic D, Hampel H (2011). Amyloid β (Aβ) and phospho-tau (p-tau) as diagnostic biomarkers in Alzheimer’s disease. Clin Chem Lab Med.

[15] Mormino EC, Papp KV (2018). Amyloid Accumulation and Cognitive decline in clinically normal older individuals: implications for aging and early Alzheimer’s Disease. J Alzheimers Dis.

[16] Wang S, Mims PN, Roman RJ, Fan F. Is Beta-Amyloid Accumulation a Cause or Consequence of Alzheimer’s Disease? J Alzheimers Parkinsonism Dement. 2016;1(2). PMC5555607 28815226

[17] Ito S, Yagi R, Ogata S, Masuda T, Saito T, Saido T, Ohtsuki S (2023). Proteomic alterations in the brain and blood–brain barrier during brain Aβ accumulation in an APP knock-in mouse model of Alzheimer’s disease. Fluids Barriers CNS.

[18] Zhou Z-d, Chan CH-s, Ma Q-h, Xu X-h, Xiao Z-c, Tan E-K (2011). The roles of amyloid precursor protein (APP) in neurogenesis. Cell Adhes Migr.

[19] Karisetty BC, Bhatnagar A, Armour EM, Beaver M, Zhang H, Elefant F. Amyloid-β peptide impact on synaptic function and neuroepigenetic Gene Control Reveal New Therapeutic Strategies for Alzheimer’s Disease. Front Mol Neurosci. 2020;13. 10.3389/fnmol.2020.577622. 10.3389/fnmol.2020.577622 PMC7693454 33304239

[20] Azargoonjahromi A (2023). Dual role of nitric oxide in Alzheimer’s disease. Nitric Oxide.

[21] O’Brien RJ, Wong PC (2011). Amyloid precursor protein processing and Alzheimer’s disease. Annu Rev Neurosci.

[22] Kojro E, Fahrenholz F (2005). The non-amyloidogenic pathway: structure and function of alpha-secretases. Subcell Biochem.

[23] Suri K, Ramesh M, Bhandari M, Gupta V, Kumar V, Govindaraju T, Murugan NA. Role of amyloidogenic and non-amyloidogenic protein spaces in neurodegenerative diseases and their mitigation using Theranostic agents. ChemBioChem. 2024:e202400224. 10.1002/cbic.202400224 38668376

[24] de Paula VJR, Guimarães FM, Diniz BS, Forlenza OV (2009). Neurobiological pathways to Alzheimer’s disease: Amyloid-beta, TAU protein or both?. Dement Neuropsychol.

[25] Azargoonjahromi A. Immunotherapy in Alzheimer’s disease: focusing on the efficacy of gantenerumab on amyloid-β clearance and cognitive decline. J Pharm Pharmacol. 2024;rgae066. 10.1093/jpp/rgae066. 10.1093/jpp/rgae066 38767981

[26] Sun X, Chen W-D, Wang Y-D. β-Amyloid: the key peptide in the pathogenesis of Alzheimer’s Disease. Front Pharmacol. 2015;6. 10.3389/fphar.2015.00221. 10.3389/fphar.2015.00221 PMC4588032 26483691

[27] Hampel H, Hardy J, Blennow K, Chen C, Perry G, Kim SH (2021). The Amyloid-β pathway in Alzheimer’s Disease. Mol Psychiatry.

[28] Haass C, Schlossmacher MG, Hung AY, Vigo-Pelfrey C, Mellon A, Ostaszewski BL (1992). Amyloid beta-peptide is produced by cultured cells during normal metabolism. Nature.

[29] Busciglio J, Gabuzda DH, Matsudaira P, Yankner BA (1993). Generation of beta-amyloid in the secretory pathway in neuronal and nonneuronal cells. Proc Natl Acad Sci U S A.

[30] Chen M, Inestrosa NC, Ross GS, Fernandez HL (1995). Platelets are the primary source of amyloid beta-peptide in human blood. Biochem Biophys Res Commun.

[31] Weaver DF (2020). Amyloid beta is an early responder cytokine and immunopeptide of the innate immune system. Alzheimers Dement (N Y).

[32] Frackowiak J, Miller DL, Potempska A, Sukontasup T, Mazur-Kolecka B (2003). Secretion and Accumulation of Aβ by brain vascular smooth muscle cells from AβPP-Swedish transgenic mice. J Neuropathology Experimental Neurol.

[33] Brothers HM, Gosztyla ML, Robinson SR (2018). The physiological roles of Amyloid-β peptide hint at New Ways to treat Alzheimer’s Disease. Front Aging Neurosci.

[34] Nhan HS, Chiang K, Koo EH (2015). The multifaceted nature of amyloid precursor protein and its proteolytic fragments: friends and foes. Acta Neuropathol.

[35] Sun X, Chen WD, Wang YD (2015). β-Amyloid: the key peptide in the pathogenesis of Alzheimer’s disease. Front Pharmacol.

[36] Adeniji AO, Adams PW, Mody VV. Chapter 7 - amyloid β hypothesis in the development of Therapeutic agents for Alzheimer’s Disease. In: Adejare A, editor. Drug Discovery approaches for the treatment of neurodegenerative disorders. Academic; 2017. pp. 109–43.

[37] Zhang X, Song W (2013). The role of APP and BACE1 trafficking in APP processing and amyloid-β generation. Alzheimers Res Ther.

[38] Rodríguez-Manotas M, Amorín-Díaz M, Cabezas-Herrera J, Acedo-Martínez A, Llorca-Escuín I (2012). Are γ-secretase and its associated Alzheimer’s disease γ problems?. Med Hypotheses.

[39] Zhang H, Wei W, Zhao M, Ma L, Jiang X, Pei H (2021). Interaction between Aβ and tau in the pathogenesis of Alzheimer’s Disease. Int J Biol Sci.

[40] Lisa K, Alexander JR, David HC, Jaya P. Activation of Ras-ERK Signaling and GSK-3 by Amyloid Precursor Protein and Amyloid Beta Facilitates Neurodegeneration in Alzheimer’s Disease. eneuro. 2017;4(2):ENEURO.0149-16.2017. 10.1523/ENEURO.0149-16.2017. 10.1523/ENEURO.0149-16.2017 PMC5367084 28374012

[41] Lee JK, Kim N-J (2017). Recent advances in the inhibition of p38 MAPK as a potential strategy for the treatment of Alzheimer’s Disease. Molecules.

[42] Selkoe DJ, Hardy J (2016). The amyloid hypothesis of Alzheimer’s disease at 25 years. EMBO Mol Med.

[43] Huang Y, Potter R, Sigurdson W, Santacruz A, Shih S, Ju Y-E (2012). Effects of age and amyloid deposition on Aβ dynamics in the human central nervous system. Arch Neurol.

[44] Portelius E, Andreasson U, Ringman JM, Buerger K, Daborg J, Buchhave P (2010). Distinct cerebrospinal fluid amyloid β peptide signatures in sporadic and PSEN1A431E-associated familial Alzheimer’s disease. Mol Neurodegeneration.

[45] Mehta PD, Pirttilä T, Mehta SP, Sersen EA, Aisen PS, Wisniewski HM (2000). Plasma and cerebrospinal fluid levels of amyloid β proteins 1–40 and 1–42 in Alzheimer disease. Arch Neurol.

[46] Iwatsubo T, Odaka A, Suzuki N, Mizusawa H, Nukina N, Ihara Y (1994). Visualization of Aβ42 (43) and Aβ40 in senile plaques with end-specific Aβ monoclonals: evidence that an initially deposited species is Aβ42 (43). Neuron.

[47] Mak K, Yang F, Vinters HV, Frautschy SA, Cole GM (1994). Polyclonals to β-amyloid (1–42) identify most plaque and vascular deposits in Alzheimer cortex, but not striatum. Brain Res.

[48] Miller DL, Papayannopoulos IA, Styles J, Bobin SA, Lin YY, Biemann K, Iqbal K (1993). Peptide compositions of the cerebrovascular and senile plaque core amyloid deposits of Alzheimer′ s disease. Arch Biochem Biophys.

[49] Azargoonjahromi A, Abutalebian F. Unraveling the Therapeutic Efficacy of Resveratrol in Alzheimer’s Disease: An Umbrella Review of Systematic Evidence. Authorea Preprints. 2023. 10.1186/s12986-024-00792-1 PMC10953289 38504306

[50] Tönnies E, Trushina E, Oxidative, Stress (2017). Synaptic dysfunction, and Alzheimer’s Disease. J Alzheimers Dis.

[51] Guo T, Zhang D, Zeng Y, Huang TY, Xu H, Zhao Y (2020). Molecular and cellular mechanisms underlying the pathogenesis of Alzheimer’s disease. Mol Neurodegeneration.

[52] Long JM, Holtzman DM (2019). Alzheimer Disease: an update on pathobiology and treatment strategies. Cell.

[53] Castellani RJ, Peclovits A, Perry G, McManus LM, Mitchell RN (2014). Neuropathology of Alzheimer’s Disease. Pathobiology of Human Disease.

[54] Kontush A (2001). Amyloid-beta: an antioxidant that becomes a pro-oxidant and critically contributes to Alzheimer’s disease. Free Radic Biol Med.

[55] Chan AC, Dharmarajan AA, Atwood CS, Huang X, Tanzi RE, Bush AI, Martins RN (1999). Anti-apoptotic action of Alzheimer Aβ. Alzheimer’s Rep.

[56] Kontush A, Berndt C, Weber W, Akopyan V, Arlt S, Schippling S, Beisiegel U (2001). Amyloid-beta is an antioxidant for lipoproteins in cerebrospinal fluid and plasma. Free Radic Biol Med.

[57] Zou K, Gong JS, Yanagisawa K, Michikawa M (2002). A novel function of monomeric amyloid beta-protein serving as an antioxidant molecule against metal-induced oxidative damage. J Neurosci.

[58] Lönnrot K, Metsä-Ketelä T, Molnár G, Ahonen JP, Latvala M, Peltola J (1996). The effect of ascorbate and ubiquinone supplementation on plasma and CSF total antioxidant capacity. Free Radic Biol Med.

[59] Atwood CS, Obrenovich ME, Liu T, Chan H, Perry G, Smith MA, Martins RN (2003). Amyloid-beta: a chameleon walking in two worlds: a review of the trophic and toxic properties of amyloid-beta. Brain Res Brain Res Rev.

[60] Plant LD, Boyle JP, Smith IF, Peers C, Pearson HA (2003). The production of amyloid beta peptide is a critical requirement for the viability of central neurons. J Neurosci.

[61] López-Toledano MA, Shelanski ML (2004). Neurogenic effect of beta-amyloid peptide in the development of neural stem cells. J Neurosci.

[62] Clemens JA, Stephenson DT (1992). Implants containing beta-amyloid protein are not neurotoxic to young and old rat brain. Neurobiol Aging.

[63] Games D, Khan KM, Soriano FG, Keim PS, Davis DL, Bryant K, Lieberburg I (1992). Lack of Alzheimer pathology after beta-amyloid protein injections in rat brain. Neurobiol Aging.

[64] McKee AC, Kowall NW, Schumacher JS, Beal MF (1998). The neurotoxicity of amyloid beta protein in aged primates. Amyloid.

[65] Leuner K, Schütt T, Kurz C, Eckert SH, Schiller C, Occhipinti A (2012). Mitochondrion-derived reactive oxygen species lead to enhanced amyloid beta formation. Antioxid Redox Signal.

[66] Sinha M, Bhowmick P, Banerjee A, Chakrabarti S (2013). Antioxidant role of amyloid β protein in cell-free and biological systems: implication for the pathogenesis of Alzheimer disease. Free Radic Biol Med.

[67] Giuffrida ML, Caraci F, Pignataro B, Cataldo S, De Bona P, Bruno V (2009). Beta-amyloid monomers are neuroprotective. J Neurosci.

[68] Niidome T, Goto Y, Kato M, Wang PL, Goh S, Tanaka N (2009). Non-fibrillar amyloid-beta peptide reduces NMDA-induced neurotoxicity, but not AMPA-induced neurotoxicity. Biochem Biophys Res Commun.

[69] Lantz MJ, Roberts AM, Delgado DD, Nichols RA (2023). The neuroprotective N-terminal amyloid-β core hexapeptide reverses reactive gliosis and gliotoxicity in Alzheimer’s disease pathology models. J Neuroinflamm.

[70] Wu J, Anwyl R, Rowan MJ (1995). beta-amyloid selectively augments NMDA receptor-mediated synaptic transmission in rat hippocampus. NeuroReport.

[71] Wu J, Anwyl R, Rowan MJ (1995). beta-Amyloid-(1–40) increases long-term potentiation in rat hippocampus in vitro. Eur J Pharmacol.

[72] Koudinov AR, Koudinova NV (2003). Amyloid beta protein restores hippocampal long term potentiation: a central role for cholesterol. Neurobiol Lipids.

[73] Tu S, Okamoto S, Lipton SA, Xu H (2014). Oligomeric Aβ-induced synaptic dysfunction in Alzheimer’s disease. Mol Neurodegener.

[74] Puzzo D, Privitera L, Leznik E, Fà M, Staniszewski A, Palmeri A, Arancio O (2008). Picomolar amyloid-beta positively modulates synaptic plasticity and memory in hippocampus. J Neurosci.

[75] Puzzo D, Privitera L, Fa M, Staniszewski A, Hashimoto G, Aziz F (2011). Endogenous amyloid-β is necessary for hippocampal synaptic plasticity and memory. Ann Neurol.

[76] Garcia-Osta A, Alberini CM (2009). Amyloid beta mediates memory formation. Learn Mem.

[77] Puzzo D, Privitera L, Palmeri A (2012). Hormetic effect of amyloid-β peptide in synaptic plasticity and memory. Neurobiol Aging.

[78] Huang Q, Liao C, Ge F, Ao J, Liu T (2022). Acetylcholine bidirectionally regulates learning and memory. J Neurorestoratology.

[79] Morley JE, Farr SA, Banks WA, Johnson SN, Yamada KA, Xu L (2010). A physiological role for amyloid-beta protein:enhancement of learning and memory. J Alzheimers Dis.

[80] Haam J, Yakel JL (2017). Cholinergic modulation of the hippocampal region and memory function. J Neurochem.

[81] Fedele E, Rivera D, Marengo B, Pronzato MA, Ricciarelli R (2015). Amyloid β: walking on the dark side of the moon. Mech Ageing Dev.

[82] Puzzo D, Gulisano W, Arancio O, Palmeri A (2015). The keystone of Alzheimer pathogenesis might be sought in Aβ physiology. Neuroscience.

[83] Ricciarelli R, Fedele E (2017). The amyloid Cascade Hypothesis in Alzheimer’s Disease: it’s time to change our mind. Curr Neuropharmacol.

[84] Dickstein DL, Walsh J, Brautigam H, Stockton SD, Gandy S, Hof PR (2010). Role of vascular risk factors and vascular dysfunction in Alzheimer’s disease. Mt Sinai J Med.

[85] Jefferies WA, Price KA, Biron KE, Fenninger F, Pfeifer CG, Dickstein DL (2013). Adjusting the compass: new insights into the role of angiogenesis in Alzheimer’s disease. Alzheimers Res Ther.

[86] Carmeliet P (2000). Mechanisms of angiogenesis and arteriogenesis. Nat Med.

[87] Atwood CS, Bowen RL, Smith MA, Perry G (2003). Cerebrovascular requirement for sealant, anti-coagulant and remodeling molecules that allow for the maintenance of vascular integrity and blood supply. Brain Res Brain Res Rev.

[88] Ristori E, Donnini S, Ziche M (2020). New insights into blood-brain barrier maintenance: the homeostatic role of β-Amyloid precursor protein in cerebral vasculature. Front Physiol.

[89] Atwood CS, Bishop GM, Perry G, Smith MA (2002). Amyloid-beta: a vascular sealant that protects against hemorrhage?. J Neurosci Res.

[90] Pfeifer LA, White LR, Ross GW, Petrovitch H, Launer LJ (2002). Cerebral amyloid angiopathy and cognitive function: the HAAS autopsy study. Neurology.

[91] Balin BJ, Gérard HC, Arking EJ, Appelt DM, Branigan PJ, Abrams JT (1998). Identification and localization of Chlamydia pneumoniae in the Alzheimer’s brain. Med Microbiol Immunol.

[92] MacIntyre A, Hammond CJ, Little CS, Appelt DM, Balin BJ (2002). Chlamydia pneumoniae infection alters the junctional complex proteins of human brain microvascular endothelial cells. FEMS Microbiol Lett.

[93] Cantara S, Donnini S, Morbidelli L, Giachetti A, Schulz R, Memo M, Ziche M (2004). Physiological levels of amyloid peptides stimulate the angiogenic response through FGF-2. Faseb j.

[94] Paris D, Townsend K, Quadros A, Humphrey J, Sun J, Brem S (2004). Inhibition of angiogenesis by abeta peptides. Angiogenesis.

[95] Friesel R, Maciag T (1999). Fibroblast growth factor prototype release and fibroblast growth factor receptor signaling. Thromb Haemost.

[96] Araki S, Shimada Y, Kaji K, Hayashi H (1990). Apoptosis of vascular endothelial cells by fibroblast growth factor deprivation. Biochem Biophys Res Commun.

[97] Luna S, Cameron DJ, Ethell DW (2013). Amyloid-β and APP deficiencies cause severe cerebrovascular defects: important work for an old villain. PLoS ONE.

[98] Jeong H, Shin H, Hong S, Kim Y (2022). Physiological roles of Monomeric Amyloid-β and implications for Alzheimer’s disease therapeutics. Exp Neurobiol.

[99] Paris D, Ait-Ghezala G, Mathura VS, Patel N, Quadros A, Laporte V, Mullan M (2005). Anti-angiogenic activity of the mutant Dutch A(beta) peptide on human brain microvascular endothelial cells. Brain Res Mol Brain Res.

[100] Patel NS, Quadros A, Brem S, Wotoczek-Obadia M, Mathura VS, Laporte V (2008). Potent anti-angiogenic motifs within the Alzheimer beta-amyloid peptide. Amyloid.

[101] Olofsson A, Sauer-Eriksson AE, Ohman A (2006). The solvent protection of alzheimer amyloid-beta-(1–42) fibrils as determined by solution NMR spectroscopy. J Biol Chem.

[102] Cameron DJ, Galvin C, Alkam T, Sidhu H, Ellison J, Luna S, Ethell DW (2012). Alzheimer’s-related peptide amyloid-β plays a conserved role in angiogenesis. PLoS ONE.

[103] Chow VW, Mattson MP, Wong PC, Gleichmann M (2010). An overview of APP processing enzymes and products. Neuromolecular Med.

[104] Capone R, Tiwari A, Hadziselimovic A, Peskova Y, Hutchison JM, Sanders CR, Kenworthy AK (2021). The C99 domain of the amyloid precursor protein resides in the disordered membrane phase. J Biol Chem.

[105] Pauwels K, Williams TL, Morris KL, Jonckheere W, Vandersteen A, Kelly G (2012). Structural basis for increased toxicity of pathological Aβ42:Aβ40 ratios in Alzheimer Disease*. J Biol Chem.

[106] Yan Y, Wang C (2006). Aβ42 is more rigid than Aβ40 at the C terminus: implications for Aβ aggregation and toxicity. J Mol Biol.

[107] Haass C, Selkoe DJ (2007). Soluble protein oligomers in neurodegeneration: lessons from the Alzheimer’s amyloid β-peptide. Nat Rev Mol Cell Biol.

[108] Wirths O, Multhaup G, Czech C, Blanchard V, Moussaoui S, Tremp G (2001). Intraneuronal Aβ accumulation precedes plaque formation in β-amyloid precursor protein and presenilin-1 double-transgenic mice. Neurosci Lett.

[109] Gómez-Ramos P, Asuncion Moran M (2007). Ultrastructural localization of intraneuronal Aβ-peptide in Alzheimer disease brains. J Alzheimers Dis.

[110] Wegiel J, Kuchna I, Nowicki K, Frackowiak J, Mazur-Kolecka B, Imaki H (2007). Intraneuronal Aβ immunoreactivity is not a predictor of brain amyloidosis-β or neurofibrillary degeneration. Acta Neuropathol.

[111] Kurochkin IV, Goto S (1994). Alzheimer’s β-amyloid peptide specifically interacts with and is degraded by insulin degrading enzyme. FEBS Lett.

[112] Iwata N, Tsubuki S, Takaki Y, Watanabe K, Sekiguchi M, Hosoki E (2000). Identification of the major Aβ1–42-degrading catabolic pathway in brain parenchyma: suppression leads to biochemical and pathological deposition. Nat Med.

[113] Huse JT, Liu K, Pijak DS, Carlin D, Lee VM-Y, Doms RW (2002). β-secretase processing in the trans-golgi network preferentially generates truncated amyloid species that accumulate in Alzheimer’s disease brain. J Biol Chem.

[114] Mueller-Steiner S, Zhou Y, Arai H, Roberson ED, Sun B, Chen J (2006). Antiamyloidogenic and neuroprotective functions of cathepsin B: implications for Alzheimer’s disease. Neuron.

[115] Schilling S, Zeitschel U, Hoffmann T, Heiser U, Francke M, Kehlen A (2008). Glutaminyl cyclase inhibition attenuates pyroglutamate Aβ and Alzheimer’s disease–like pathology. Nat Med.

[116] Fernández-Gamba A, Leal M, Morelli L, Castaño E (2009). Insulin-degrading enzyme: structure-function relationship and its possible roles in Health and Disease. Curr Pharm Design.

[117] Farris W, Mansourian S, Chang Y, Lindsley L, Eckman EA, Frosch MP (2003). Insulin-degrading enzyme regulates the levels of insulin, amyloid beta-protein, and the beta-amyloid precursor protein intracellular domain in vivo. Proc Natl Acad Sci U S A.

[118] Tian Y, Jing G, Zhang M (2023). Insulin-degrading enzyme: roles and pathways in ameliorating cognitive impairment associated with Alzheimer’s disease and diabetes. Ageing Res Rev.

[119] Corraliza-Gomez M, Bermejo T, Lilue J, Rodriguez-Iglesias N, Valero J, Cozar-Castellano I (2023). Insulin-degrading enzyme (IDE) as a modulator of microglial phenotypes in the context of Alzheimer’s disease and brain aging. J Neuroinflamm.

[120] Kullenberg H, Nyström T, Kumlin M, Svedberg MM (2023). Correlation between insulin-degrading enzyme versus total tau and selected cytokines in patients with Alzheimer´s disease compared to non-demented controls. Neuro Endocrinol Lett.

[121] Guo H, Cheng Y, Wu J, Wang C, Wang H, Zhang C (2015). Donepezil improves learning and memory deficits in APP/PS1 mice by inhibition of microglial activation. Neuroscience.

[122] Edland S, Vriesé F, Compton D, Smith G, Ivnik R, Boeve B (2003). Insulin degrading enzyme (IDE) genetic variants and risk of Alzheimer’s disease: evidence of effect modification by apolipoprotein E (APOE). Neurosci Lett.

[123] Edland SD (2004). Insulin-degrading enzyme, apolipoprotein E, and Alzheimer’s disease. J Mol Neurosci.

[124] Nalivaeva NN, Turner AJ. Chapter 127 - Neprilysin. In: Rawlings ND, Salvesen G, editors. Handbook of Proteolytic Enzymes (Third Edition). Academic Press; 2013. pp. 612-9.

[125] Nalivaeva NN, Zhuravin IA, Turner AJ (2020). Neprilysin expression and functions in development, ageing and disease. Mech Ageing Dev.

[126] Schoenfeld HA, West T, Verghese PB, Holubasch M, Shenoy N, Kagan D (2017). The effect of angiotensin receptor neprilysin inhibitor, sacubitril/valsartan, on central nervous system amyloid-β concentrations and clearance in the cynomolgus monkey. Toxicol Appl Pharmcol.

[127] Hama E, Shirotani K, Iwata N, Saido TC (2004). Effects of Neprilysin Chimeric Proteins Targeted to subcellular compartments on amyloid β peptide clearance in primary Neurons*. J Biol Chem.

[128] Sikanyika NL, Parkington HC, Smith AI, Kuruppu S (2019). Powering amyloid beta degrading enzymes: a possible therapy for Alzheimer’s disease. Neurochem Res.

[129] Becker M, Moore A, Naughton M, Boland B, Siems W-E, Walther T (2018). Neprilysin degrades murine amyloid-β (Aβ) more efficiently than human Aβ: further implication for species-specific amyloid accumulation. Neurosci Lett.

[130] Rofo F, Metzendorf NG, Saubi C, Suominen L, Godec A, Sehlin D (2022). Blood–brain barrier penetrating neprilysin degrades monomeric amyloid-beta in a mouse model of Alzheimer’s disease. Alzheimers Res Ther.

[131] El-Amouri SS, Zhu H, Yu J, Marr R, Verma IM, Kindy MS (2008). Neprilysin: an enzyme candidate to slow the progression of Alzheimer’s disease. Am J Pathol.

[132] Oberstein TJ, Utz J, Spitzer P, Klafki HW, Wiltfang J, Lewczuk P (2020). The role of cathepsin B in the degradation of Aβ and in the production of Aβ peptides starting with Ala2 in cultured astrocytes. Front Mol Neurosci.

[133] Oberstein TJ, Utz J, Spitzer P, Klafki HW, Wiltfang J, Lewczuk P (2021). The role of cathepsin B in the degradation of Aβ and in the production of Aβ peptides starting with Ala2 in cultured astrocytes. Front Mol Neurosci.

[134] Shimizu T, Fukuda H, Murayama S, Izumiyama N, Shirasawa T (2002). Isoaspartate formation at position 23 of amyloid beta peptide enhanced fibril formation and deposited onto senile plaques and vascular amyloids in Alzheimer’s disease. J Neurosci Res.

[135] Gnoth K, Piechotta A, Kleinschmidt M, Konrath S, Schenk M, Taudte N (2020). Targeting isoaspartate-modified Aβ rescues behavioral deficits in transgenic mice with Alzheimer’s disease-like pathology. Alzheimers Res Ther.

[136] Shimizu T, Watanabe A, Ogawara M, Mori H, Shirasawa T (2000). Isoaspartate formation and neurodegeneration in Alzheimer’s Disease. Arch Biochem Biophys.

[137] Wang J, Guo C, Meng Z, Zwan MD, Chen X, Seelow S (2023). Testing the link between isoaspartate and Alzheimer’s disease etiology. Alzheimer’s Dement.

[138] Shaw LM, Vanderstichele H, Knapik-Czajka M, Clark CM, Aisen PS, Petersen RC (2009). Cerebrospinal fluid biomarker signature in Alzheimer’s disease neuroimaging initiative subjects. Ann Neurol.

[139] Walsh DM, Klyubin I, Fadeeva JV, Cullen WK, Anwyl R, Wolfe MS (2002). Naturally secreted oligomers of amyloid β protein potently inhibit hippocampal long-term potentiation in vivo. Nature.

[140] Lambert MP, Barlow AK, Chromy BA, Edwards C, Freed R, Liosatos M (1998). Diffusible, nonfibrillar ligands derived from Abeta1-42 are potent central nervous system neurotoxins. Proc Natl Acad Sci U S A.

[141] Cheng IH, Scearce-Levie K, Legleiter J, Palop JJ, Gerstein H, Bien-Ly N (2007). Accelerating amyloid-beta fibrillization reduces oligomer levels and functional deficits in Alzheimer disease mouse models. J Biol Chem.

[142] Hsia AY, Masliah E, McConlogue L, Yu GQ, Tatsuno G, Hu K (1999). Plaque-independent disruption of neural circuits in Alzheimer’s disease mouse models. Proc Natl Acad Sci U S A.

[143] Meilandt WJ, Cisse M, Ho K, Wu T, Esposito LA, Scearce-Levie K (2009). Neprilysin overexpression inhibits plaque formation but fails to reduce pathogenic abeta oligomers and associated cognitive deficits in human amyloid precursor protein transgenic mice. J Neurosci.

[144] Mc Donald JM, Savva GM, Brayne C, Welzel AT, Forster G, Shankar GM (2010). The presence of sodium dodecyl sulphate-stable Aβ dimers is strongly associated with Alzheimer-type dementia. Brain.

[145] McLean CA, Cherny RA, Fraser FW, Fuller SJ, Smith MJ, Vbeyreuther K (1999). Soluble pool of Aβ amyloid as a determinant of severity of neurodegeneration in Alzheimer’s disease. Ann Neurol.

[146] Texidó L, Martín-Satué M, Alberdi E, Solsona C, Matute C (2011). Amyloid β peptide oligomers directly activate NMDA receptors. Cell Calcium.

[147] Taniguchi K, Yamamoto F, Amano A, Tamaoka A, Sanjo N, Yokota T (2022). Amyloid-β oligomers interact with NMDA receptors containing GluN2B subunits and metabotropic glutamate receptor 1 in primary cortical neurons: relevance to the synapse pathology of Alzheimer’s disease. Neurosci Res.

[148] Liu J, Chang L, Song Y, Li H, Wu Y. The role of NMDA receptors in Alzheimer’s Disease. Front NeuroSci. 2019;13. 10.3389/fnins.2019.00043. 10.3389/fnins.2019.00043 PMC6375899 30800052

[149] Palop JJ, Mucke L (2010). Amyloid-beta-induced neuronal dysfunction in Alzheimer’s disease: from synapses toward neural networks. Nat Neurosci.

[150] Selkoe DJ (2000). Toward a comprehensive theory for Alzheimer’s disease. Hypothesis: Alzheimer’s disease is caused by the cerebral accumulation and cytotoxicity of amyloid beta-protein. Ann N Y Acad Sci.

[151] Shankar GM, Li S, Mehta TH, Garcia-Munoz A, Shepardson NE, Smith I (2008). Amyloid-beta protein dimers isolated directly from Alzheimer’s brains impair synaptic plasticity and memory. Nat Med.

[152] Butterfield DA, Swomley AM, Sultana R (2013). Amyloid β-peptide (1–42)-induced oxidative stress in Alzheimer disease: importance in disease pathogenesis and progression. Antioxid Redox Signal.

[153] Fani G, Mannini B, Vecchi G, Cascella R, Cecchi C, Dobson CM (2021). Aβ oligomers Dysregulate Calcium Homeostasis by Mechanosensitive activation of AMPA and NMDA receptors. ACS Chem Neurosci.

[154] Ow SY, Dunstan DE (2014). A brief overview of amyloids and Alzheimer’s disease. Protein Sci.

[155] Chen GF, Xu TH, Yan Y, Zhou YR, Jiang Y, Melcher K, Xu HE (2017). Amyloid beta: structure, biology and structure-based therapeutic development. Acta Pharmacol Sin.

[156] Miao J, Ma H, Yang Y, Liao Y, Lin C, Zheng J, et al. Microglia in Alzheimer’s disease: pathogenesis, mechanisms, and therapeutic potentials. Front Aging Neurosci. 2023;15. 10.3389/fnagi.2023.1201982. 10.3389/fnagi.2023.1201982 PMC10309009 37396657

[157] Ries M, Sastre M (2016). Mechanisms of Aβ clearance and degradation by glial cells. Front Aging Neurosci.

[158] Salvadores N, Moreno-Gonzalez I, Gamez N, Quiroz G, Vegas-Gomez L, Escandón M (2022). Aβ oligomers trigger necroptosis-mediated neurodegeneration via microglia activation in Alzheimer’s disease. Acta Neuropathol Commun.

[159] Mohamed A, Posse de Chaves E (2011). A < i > β internalization by neurons and Glia. Int J Alzheimer’s Disease.

[160] Jarosz-Griffiths HH, Noble E, Rushworth JV, Hooper NM (2016). Amyloid-β receptors: the Good, the bad, and the prion Protein*. J Biol Chem.

[161] Tolar M, Hey J, Power A, Abushakra S. Neurotoxic Soluble Amyloid Oligomers Drive Alzheimer’s Pathogenesis and Represent a Clinically Validated Target for Slowing Disease Progression. Int J Mol Sci. 2021;22(12). 10.3390/ijms22126355. 10.3390/ijms22126355 PMC8231952 34198582

[162] Sun X, Dong S, Kato H, Kong J, Ito Y, Hirofuji Y (2022). Mitochondrial calcium-triggered oxidative stress and developmental defects in dopaminergic neurons differentiated from deciduous teeth-derived Dental Pulp Stem cells with MFF Insufficiency. Antioxidants.

[163] Zündorf G, Reiser G (2011). Calcium dysregulation and homeostasis of neural calcium in the molecular mechanisms of neurodegenerative diseases provide multiple targets for neuroprotection. Antioxid Redox Signal.

[164] Arghavani P, Pirhaghi M, Moosavi-Movahedi F, Mamashli F, Hosseini E, Moosavi-Movahedi AA (2022). Amyloid management by chaperones: the mystery underlying protein oligomers’ dual functions. Curr Res Struct Biol.

[165] Smith HL, Li W, Cheetham ME (2015). Molecular chaperones and neuronal proteostasis. Semin Cell Dev Biol.

[166] Wentink A, Nussbaum-Krammer C, Bukau B (2019). Modulation of amyloid states by molecular chaperones. Cold Spring Harb Perspect Biol.

[167] Sakono M, Zako T (2010). Amyloid oligomers: formation and toxicity of Aβ oligomers. FEBS J.

[168] Zhang Y, Chen X, Zhao Y, Ponnusamy M, Liu Y (2017). The role of ubiquitin proteasomal system and autophagy-lysosome pathway in Alzheimer’s disease. Rev Neurosci.

[169] Tseng BP, Green KN, Chan JL, Blurton-Jones M, LaFerla FM (2008). Abeta inhibits the proteasome and enhances amyloid and tau accumulation. Neurobiol Aging.

[170] de la Cueva M, Antequera D, Ordoñez-Gutierrez L, Wandosell F, Camins A, Carro E, Bartolome F (2022). Amyloid-β impairs mitochondrial dynamics and autophagy in Alzheimer’s disease experimental models. Sci Rep.

[171] Zhang W, Xu C, Sun J, Shen H-M, Wang J, Yang C (2022). Impairment of the autophagy–lysosomal pathway in Alzheimer’s diseases: pathogenic mechanisms and therapeutic potential. Acta Pharm Sinica B.

[172] Parameshwaran K, Dhanasekaran M, Suppiramaniam V (2008). Amyloid beta peptides and glutamatergic synaptic dysregulation. Exp Neurol.

[173] Butterfield DA, Reed T, Newman SF, Sultana R (2007). Roles of amyloid β-peptide-associated oxidative stress and brain protein modifications in the pathogenesis of Alzheimer’s disease and mild cognitive impairment. Free Radic Biol Med.

[174] Rosales-Corral S, Tan D-X, Reiter RJ, Valdivia-Velázquez M, Acosta-Martínez JP, Ortiz GG (2004). Kinetics of the neuroinflammation-oxidative stress correlation in rat brain following the injection of fibrillar amyloid-β onto the hippocampus in vivo. J Neuroimmunol.

[175] Lin H, Bhatia R, Lal R (2001). Amyloid β protein forms ion channels: implications for Alzheimer’s disease pathophysiology. FASEB J.

[176] Canevari L, Clark JB, Bates TE (1999). β-Amyloid fragment 25–35 selectively decreases complex IV activity in isolated mitochondria. FEBS Lett.

[177] Varadarajan S, Yatin S, Aksenova M, Butterfield DA, Review (2000). Alzheimer’s amyloid beta-peptide-associated free radical oxidative stress and neurotoxicity. J Struct Biol.

[178] Smith DG, Cappai R, Barnham KJ (2007). The redox chemistry of the Alzheimer’s disease amyloid beta peptide. Biochim Biophys Acta.

[179] Hureau C, Faller P (2009). Abeta-mediated ROS production by Cu ions: structural insights, mechanisms and relevance to Alzheimer’s disease. Biochimie.

[180] Rottkamp CA, Raina AK, Zhu X, Gaier E, Bush AI, Atwood CS (2001). Redox-active iron mediates amyloid-beta toxicity. Free Radic Biol Med.

[181] Butterfield DA, Boyd-Kimball D (2005). The critical role of methionine 35 in Alzheimer’s amyloid beta-peptide (1–42)-induced oxidative stress and neurotoxicity. Biochim Biophys Acta.

[182] Dong J, Atwood CS, Anderson VE, Siedlak SL, Smith MA, Perry G, Carey PR (2003). Metal binding and oxidation of amyloid-beta within isolated senile plaque cores: Raman microscopic evidence. Biochemistry.

[183] Dasilva KA, Shaw JE, McLaurin J (2010). Amyloid-beta fibrillogenesis: structural insight and therapeutic intervention. Exp Neurol.

[184] Butterfield DA, Reed T, Newman SF, Sultana R (2007). Roles of amyloid beta-peptide-associated oxidative stress and brain protein modifications in the pathogenesis of Alzheimer’s disease and mild cognitive impairment. Free Radic Biol Med.

[185] Wan L, Nie G, Zhang J, Luo Y, Zhang P, Zhang Z, Zhao B (2011). β-Amyloid peptide increases levels of iron content and oxidative stress in human cell and Caenorhabditis elegans models of Alzheimer disease. Free Radic Biol Med.

[186] Selkoe DJ (2008). Soluble oligomers of the amyloid beta-protein impair synaptic plasticity and behavior. Behav Brain Res.

[187] Reddy PH, Beal MF (2008). Amyloid beta, mitochondrial dysfunction and synaptic damage: implications for cognitive decline in aging and Alzheimer’s disease. Trends Mol Med.

[188] Oddo S, Caccamo A, Shepherd JD, Murphy MP, Golde TE, Kayed R (2003). Triple-transgenic model of Alzheimer’s disease with plaques and tangles: intracellular abeta and synaptic dysfunction. Neuron.

[189] Walsh DM, Klyubin I, Fadeeva JV, Cullen WK, Anwyl R, Wolfe MS (2002). Naturally secreted oligomers of amyloid beta protein potently inhibit hippocampal long-term potentiation in vivo. Nature.

[190] Cerpa W, Dinamarca MC, Inestrosa NC (2008). Structure-function implications in Alzheimer’s disease: effect of Abeta oligomers at central synapses. Curr Alzheimer Res.

[191] Moreno H, Yu E, Pigino G, Hernandez AI, Kim N, Moreira JE (2009). Synaptic transmission block by presynaptic injection of oligomeric amyloid beta. Proc Natl Acad Sci U S A.

[192] Pigino G, Morfini G, Atagi Y, Deshpande A, Yu C, Jungbauer L (2009). Disruption of fast axonal transport is a pathogenic mechanism for intraneuronal amyloid beta. Proc Natl Acad Sci U S A.

[193] Shipton OA, Leitz JR, Dworzak J, Acton CE, Tunbridge EM, Denk F (2011). Tau protein is required for amyloid {beta}-induced impairment of hippocampal long-term potentiation. J Neurosci.

[194] Hartz AM, Bauer B, Soldner EL, Wolf A, Boy S, Backhaus R (2012). Amyloid-β contributes to blood-brain barrier leakage in transgenic human amyloid precursor protein mice and in humans with cerebral amyloid angiopathy. Stroke.

[195] Wang D, Chen F, Han Z, Yin Z, Ge X, Lei P (2021). Relationship between Amyloid-β deposition and blood-brain barrier dysfunction in Alzheimer’s Disease. Front Cell Neurosci.

[196] Kook SY, Hong HS, Moon M, Ha CM, Chang S, Mook-Jung I (2012). Aβ₁₋₄₂-RAGE interaction disrupts tight junctions of the blood-brain barrier via Ca²⁺-calcineurin signaling. J Neurosci.

[197] Marco S, Skaper SD (2006). Amyloid beta-peptide1-42 alters tight junction protein distribution and expression in brain microvessel endothelial cells. Neurosci Lett.

[198] Claudio L (1996). Ultrastructural features of the blood-brain barrier in biopsy tissue from Alzheimer’s disease patients. Acta Neuropathol.

[199] Baier M, Apelt J, Riemer C, Gültner S, Schwarz A, Bamme T (2008). Prion infection of mice transgenic for human APPSwe: increased accumulation of cortical formic acid extractable abeta(1–42) and rapid scrapie disease development. Int J Dev Neurosci.

[200] Parkin ET, Watt NT, Hussain I, Eckman EA, Eckman CB, Manson JC (2007). Cellular prion protein regulates beta-secretase cleavage of the Alzheimer’s amyloid precursor protein. Proc Natl Acad Sci U S A.

[201] Perry VH, Holmes C (2014). Microglial priming in neurodegenerative disease. Nat Reviews Neurol.

[202] Perry VH, Holmes C (2014). Microglial priming in neurodegenerative disease. Nat Rev Neurol.

[203] Condello C, Yuan P, Schain A, Grutzendler J (2015). Microglia constitute a barrier that prevents neurotoxic protofibrillar Aβ42 hotspots around plaques. Nat Commun.

[204] Heneka MT, Carson MJ, El Khoury J, Landreth GE, Brosseron F, Feinstein DL (2015). Neuroinflammation in Alzheimer’s disease. Lancet Neurol.

[205] Wang S, Colonna M (2019). Microglia in Alzheimer’s disease: a target for immunotherapy. J Leukoc Biol.

[206] Sarlus H, Heneka MT (2017). Microglia in Alzheimer’s disease. J Clin Invest.

[207] Forloni G, Balducci C (2018). Alzheimer’s Disease, Oligomers, and inflammation. J Alzheimers Dis.

[208] Glass CK, Saijo K, Winner B, Marchetto MC, Gage FH (2010). Mechanisms underlying inflammation in neurodegeneration. Cell.

[209] Cameron B, Landreth GE (2010). Inflammation, microglia, and Alzheimer’s disease. Neurobiol Dis.

[210] Cantarella G, Di Benedetto G, Puzzo D, Privitera L, Loreto C, Saccone S (2015). Neutralization of TNFSF10 ameliorates functional outcome in a murine model of Alzheimer’s disease. Brain.

[211] Sharma D, Kanneganti TD (2016). The cell biology of inflammasomes: mechanisms of inflammasome activation and regulation. J Cell Biol.

[212] Von Bernhardi R, Cornejo F, Parada GE, Eugenín J (2015). Role of TGFβ signaling in the pathogenesis of Alzheimer’s disease. Front Cell Neurosci.

[213] Van Eldik LJ, Carrillo MC, Cole PE, Feuerbach D, Greenberg BD, Hendrix JA (2016). The roles of inflammation and immune mechanisms in Alzheimer’s disease. Alzheimer’s Dementia: Translational Res Clin Interventions.

[214] Brosseron F, Krauthausen M, Kummer M, Heneka MT (2014). Body fluid cytokine levels in mild cognitive impairment and Alzheimer’s disease: a comparative overview. Mol Neurobiol.

[215] Wyss-Coray T, Lin C, Yan F, Yu GQ, Rohde M, McConlogue L (2001). TGF-beta1 promotes microglial amyloid-beta clearance and reduces plaque burden in transgenic mice. Nat Med.

[216] Su C, Miao J, Guo J (2023). The relationship between TGF-β1 and cognitive function in the brain. Brain Res Bull.

[217] Wang W-Y, Tan M-S, Yu J-T, Tan L (2015). Role of pro-inflammatory cytokines released from microglia in Alzheimer’s disease. Annals Translational Med.

[218] Rani V, Verma R, Kumar K, Chawla R (2023). Role of pro-inflammatory cytokines in Alzheimer’s disease and neuroprotective effects of pegylated self-assembled nanoscaffolds. Curr Res Pharmacol Drug Discov.

[219] Liddelow SA, Guttenplan KA, Clarke LE, Bennett FC, Bohlen CJ, Schirmer L (2017). Neurotoxic reactive astrocytes are induced by activated microglia. Nature.

[220] Wang MM, Miao D, Cao XP, Tan L, Tan L (2018). Innate immune activation in Alzheimer’s disease. Ann Transl Med.

[221] Guerreiro R, Wojtas A, Bras J, Carrasquillo M, Rogaeva E, Majounie E (2013). TREM2 variants in Alzheimer’s disease. N Engl J Med.

[222] Lee CYD, Daggett A, Gu X, Jiang LL, Langfelder P, Li X (2018). Elevated TREM2 gene dosage reprograms Microglia Responsivity and ameliorates pathological phenotypes in Alzheimer’s Disease models. Neuron.

[223] Olabarria M, Noristani HN, Verkhratsky A, Rodríguez JJ (2010). Concomitant astroglial atrophy and astrogliosis in a triple transgenic animal model of Alzheimer’s disease. Glia.

[224] Arranz AM, De Strooper B (2019). The role of astroglia in Alzheimer’s disease: pathophysiology and clinical implications. Lancet Neurol.

[225] Wyss-Coray T, Loike JD, Brionne TC, Lu E, Anankov R, Yan F (2003). Adult mouse astrocytes degrade amyloid-beta in vitro and in situ. Nat Med.

[226] Di Benedetto G, Burgaletto C, Bellanca CM, Munafò A, Bernardini R, Cantarella G. Role of Microglia and Astrocytes in Alzheimer’s Disease: From Neuroinflammation to Ca(2+) Homeostasis Dysregulation. Cells. 2022;11(17). 10.3390/cells11172728. 10.3390/cells11172728 PMC9454513 36078138

[227] Bellaver B, Povala G, Ferreira PCL, Ferrari-Souza JP, Leffa DT, Lussier FZ (2023). Astrocyte reactivity influences amyloid-β effects on tau pathology in preclinical Alzheimer’s disease. Nat Med.

[228] Batarseh YS, Duong Q-V, Mousa YM, Al Rihani SB, Elfakhri K, Kaddoumi A (2016). Amyloid-β and astrocytes interplay in Amyloid-β related disorders. Int J Mol Sci.

[229] Davis N, Mota BC, Stead L, Palmer EOC, Lombardero L, Rodríguez-Puertas R (2021). Pharmacological ablation of astrocytes reduces Aβ degradation and synaptic connectivity in an ex vivo model of Alzheimer’s disease. J Neuroinflammation.

[230] Ries M, Sastre M. Mechanisms of Aβ clearance and degradation by glial cells. Front Aging Neurosci. 2016;8. 10.3389/fnagi.2016.00160. 10.3389/fnagi.2016.00160 PMC4932097 27458370

[231] Romeo R, Glotzbach K, Scheller A, Faissner A (2020). Deletion of LRP1 from astrocytes modifies neuronal network activity in an in vitro model of the tripartite synapse. Front Cell Neurosci.

[232] Romeo R, Boden-El Mourabit D, Scheller A, Mark MD, Faissner A. Low-density lipoprotein receptor-related protein 1 (LRP1) as a Novel Regulator of Early Astroglial differentiation. Front Cell Neurosci. 2021;15. 10.3389/fncel.2021.642521. 10.3389/fncel.2021.642521 PMC7930235 33679332

[233] van Kralingen C, Kho DT, Costa J, Angel CE, Graham ES (2013). Exposure to inflammatory cytokines IL-1β and TNFα induces compromise and death of astrocytes; implications for chronic neuroinflammation. PLoS ONE.

[234] Hyvärinen T, Hagman S, Ristola M, Sukki L, Veijula K, Kreutzer J (2019). Co-stimulation with IL-1β and TNF-α induces an inflammatory reactive astrocyte phenotype with neurosupportive characteristics in a human pluripotent stem cell model system. Sci Rep.

[235] Giovannoni F, Quintana FJ (2020). The role of astrocytes in CNS inflammation. Trends Immunol.

[236] Sheng WS, Hu S, Feng A, Rock RB (2013). Reactive oxygen species from human astrocytes induced functional impairment and oxidative damage. Neurochem Res.

[237] Rizor A, Pajarillo E, Johnson J, Aschner M, Lee E (2019). Astrocytic Oxidative/Nitrosative Stress Contributes to Parkinson’s Disease Pathogenesis: the dual role of reactive astrocytes. Antioxidants.

[238] Tran AP, Warren PM, Silver J (2022). New insights into glial scar formation after spinal cord injury. Cell Tissue Res.

[239] Frost GR, Li YM. The role of astrocytes in amyloid production and Alzheimer’s disease. Open Biol. 2017;7(12). 10.1098/rsob.170228. 10.1098/rsob.170228 PMC5746550 29237809

[240] Strickland MR, Rau MJ, Summers B, Basore K, Wulf J, Jiang H (2024). Apolipoprotein E secreted by astrocytes forms antiparallel dimers in discoidal lipoproteins. Neuron.

[241] Garland EF, Hartnell IJ, Boche D. Microglia and astrocyte function and communication: what do we know in humans? Front NeuroSci. 2022;16. 10.3389/fnins.2022.824888. 10.3389/fnins.2022.824888 PMC8888691 35250459

[242] He M, Dong H, Huang Y, Lu S, Zhang S, Qian Y, Jin W (2016). Astrocyte-derived CCL2 is Associated with M1 activation and recruitment of cultured Microglial cells. Cell Physiol Biochem.

[243] Madrigal JL, Leza JC, Polak P, Kalinin S, Feinstein DL (2009). Astrocyte-derived MCP-1 mediates neuroprotective effects of noradrenaline. J Neurosci.

[244] Thompson W, Van Eldik L (2009). Inflammatory cytokines stimulate the chemokines CCL2/MCP-1 and CCL7/MCP-7 through NFkappaB and MAPK dependent pathways in rat astrocytes. Brain Res.

[245] Motolani A, Martin M, Sun M, Lu T. Reference Module in Biomed Science. Elsevier Amsterdam, The Netherlands:; 2021.

[246] Sun SC (2011). Non-canonical NF-κB signaling pathway. Cell Res.

[247] Chiarini A, Armato U, Hu P, Dal Prà I. Danger-Sensing/Patten Recognition Receptors and Neuroinflammation in Alzheimer’s Disease. Int J Mol Sci. 2020;21(23). 10.3390/ijms21239036. 10.3390/ijms21239036 PMC7731137 33261147

[248] Thawkar BS, Kaur G (2019). Inhibitors of NF-κB and P2 × 7/NLRP3/Caspase 1 pathway in microglia: novel therapeutic opportunities in neuroinflammation induced early-stage Alzheimer’s disease. J Neuroimmunol.

[249] Lukiw WJ (2016). Bacteroides fragilis Lipopolysaccharide and Inflammatory Signaling in Alzheimer’s Disease. Front Microbiol.

[250] Zhan X, Stamova B, Sharp FR (2018). Lipopolysaccharide Associates with amyloid plaques, neurons and oligodendrocytes in Alzheimer’s Disease Brain: a review. Front Aging Neurosci.

[251] Sun E, Motolani A, Campos L, Lu T. The Pivotal Role of NF-kB in the Pathogenesis and Therapeutics of Alzheimer’s Disease. Int J Mol Sci. 2022;23(16). 10.3390/ijms23168972. 10.3390/ijms23168972 PMC9408758 36012242

[252] Lawrence T (2009). The nuclear factor NF-kappaB pathway in inflammation. Cold Spring Harb Perspect Biol.

[253] Hoesel B, Schmid JA (2013). The complexity of NF-κB signaling in inflammation and cancer. Mol Cancer.

[254] Chen CH, Zhou W, Liu S, Deng Y, Cai F, Tone M (2012). Increased NF-κB signalling up-regulates BACE1 expression and its therapeutic potential in Alzheimer’s disease. Int J Neuropsychopharmacol.

[255] Snow WM, Albensi BC (2016). Neuronal gene targets of NF-κB and their dysregulation in Alzheimer’s Disease. Front Mol Neurosci.

[256] Valerio A, Boroni F, Benarese M, Sarnico I, Ghisi V, Bresciani LG (2006). NF-kappaB pathway: a target for preventing beta-amyloid (Abeta)-induced neuronal damage and Abeta42 production. Eur J Neurosci.

[257] Behl C, Davis JB, Lesley R, Schubert D (1994). Hydrogen peroxide mediates amyloid beta protein toxicity. Cell.

[258] Gulisano W, Maugeri D, Baltrons MA, Fà M, Amato A, Palmeri A (2018). Role of Amyloid-β and tau proteins in Alzheimer’s Disease: confuting the amyloid Cascade. J Alzheimers Dis.

[259] Gong CX, Iqbal K (2008). Hyperphosphorylation of microtubule-associated protein tau: a promising therapeutic target for Alzheimer disease. Curr Med Chem.

[260] Giraldo E, Lloret A, Fuchsberger T, Viña J (2014). Aβ and tau toxicities in Alzheimer’s are linked via oxidative stress-induced p38 activation: protective role of vitamin E. Redox Biol.

[261] Lloret A, Fuchsberger T, Giraldo E, Viña J (2015). Molecular mechanisms linking amyloid β toxicity and tau hyperphosphorylation in Alzheimer׳ s disease. Free Radic Biol Med.

[262] Munoz L, Ammit AJ (2010). Targeting p38 MAPK pathway for the treatment of Alzheimer’s disease. Neuropharmacology.

[263] Kheiri G, Dolatshahi M, Rahmani F, Rezaei N (2018). Role of p38/MAPKs in Alzheimer’s disease: implications for amyloid beta toxicity targeted therapy. Rev Neurosci.

[264] Lim S, Haque MM, Kim D, Kim DJ, Kim YK (2014). Cell-based models to investigate tau aggregation. Comput Struct Biotechnol J.

[265] Li X, Chen Y, Yang Z, Zhang S, Wei G, Zhang L (2024). Structural insights into the co-aggregation of Aβ and tau amyloid core peptides: revealing potential pathological heterooligomers by simulations. Int J Biol Macromol.

[266] Di Battista AM, Heinsinger NM, Rebeck GW (2016). Alzheimer’s Disease Genetic risk factor APOE-ε4 also affects normal brain function. Curr Alzheimer Res.

[267] Liu CC, Liu CC, Kanekiyo T, Xu H, Bu G (2013). Apolipoprotein E and Alzheimer disease: risk, mechanisms and therapy. Nat Rev Neurol.

[268] Lumsden AL, Mulugeta A, Zhou A, Hyppönen E. Apolipoprotein E (APOE) genotype-associated disease risks: a phenome-wide, registry-based, case-control study utilising the UK Biobank. EBioMedicine. 2020;59. 10.1016/j.ebiom.2020.102954 PMC7452404 32818802

[269] Troutwine BR, Hamid L, Lysaker CR, Strope TA, Wilkins HM (2022). Apolipoprotein E and Alzheimer’s disease. Acta Pharm Sinica B.

[270] Yamazaki Y, Painter MM, Bu G, Kanekiyo T (2016). Apolipoprotein E as a therapeutic target in Alzheimer’s disease: a review of Basic Research and clinical evidence. CNS Drugs.

[271] Hashimoto T, Serrano-Pozo A, Hori Y, Adams KW, Takeda S, Banerji AO (2012). Apolipoprotein E, especially apolipoprotein E4, increases the oligomerization of amyloid β peptide. J Neurosci.

[272] Koffie RM, Hashimoto T, Tai H-C, Kay KR, Serrano-Pozo A, Joyner D (2012). Apolipoprotein E4 effects in Alzheimer’s disease are mediated by synaptotoxic oligomeric amyloid-β. Brain.

[273] Christensen DZ, Schneider-Axmann T, Lucassen PJ, Bayer TA, Wirths O (2010). Accumulation of intraneuronal Aβ correlates with ApoE4 genotype. Acta Neuropathol.

[274] Kok E, Haikonen S, Luoto T, Huhtala H, Goebeler S, Haapasalo H, Karhunen PJ (2009). Apolipoprotein E–dependent accumulation of Alzheimer disease–related lesions begins in middle age. Annals Neurology: Official J Am Neurol Association Child Neurol Soc.

[275] Schmechel D, Saunders A, Strittmatter W, Crain BJ, Hulette C, Joo S et al. Increased amyloid beta-peptide deposition in cerebral cortex as a consequence of apolipoprotein E genotype in late-onset Alzheimer disease. Proceedings of the National Academy of Sciences. 1993;90(20):9649-53. 10.1073/pnas.90.20.9649 PMC47627 8415756

[276] Rannikmae K, Kalaria R, Greenberg S, Chui H, Schmitt F, Samarasekera N et al. APOE allele-specific associations with severe CAA-associated vasculopathic changes-collaborative meta-analysis. UK Stroke Forum 2013 Conference: Newcastle University; 2013.

[277] Shinohara M, Murray ME, Frank RD, Shinohara M, DeTure M, Yamazaki Y (2016). Impact of sex and APOE4 on cerebral amyloid angiopathy in Alzheimer’s disease. Acta Neuropathol.

[278] Sullivan P, Mace B, Maeda N, Schmechel D (2004). Marked regional differences of brain human apolipoprotein E expression in targeted replacement mice. Neuroscience.

[279] Bales KR, Liu F, Wu S, Lin S, Koger D, DeLong C (2009). Human APOE isoform-dependent effects on brain β-amyloid levels in PDAPP transgenic mice. J Neurosci.

[280] Castellano JM, Kim J, Stewart FR, Jiang H, DeMattos RB, Patterson BW (2011). Human apoE isoforms differentially regulate brain amyloid-β peptide clearance. Sci Transl Med.

[281] Youmans KL, Tai LM, Nwabuisi-Heath E, Jungbauer L, Kanekiyo T, Gan M (2012). APOE4-specific changes in Aβ accumulation in a new transgenic mouse model of Alzheimer disease. J Biol Chem.

[282] Raulin AC, Doss SV, Trottier ZA, Ikezu TC, Bu G, Liu CC (2022). ApoE in Alzheimer’s disease: pathophysiology and therapeutic strategies. Mol Neurodegener.

[283] Holtzman DM, Herz J, Bu G (2012). Apolipoprotein E and apolipoprotein E receptors: normal biology and roles in Alzheimer disease. Cold Spring Harb Perspect Med.

[284] Friedberg JS, Aytan N, Cherry JD, Xia W, Standring OJ, Alvarez VE (2020). Associations between brain inflammatory profiles and human neuropathology are altered based on apolipoprotein E ε4 genotype. Sci Rep.

[285] Fernandez CG, Hamby ME, McReynolds ML, Ray WJ (2019). The role of APOE4 in disrupting the homeostatic functions of astrocytes and Microglia in Aging and Alzheimer’s Disease. Front Aging Neurosci.

[286] Montagne A, Nation DA, Sagare AP, Barisano G, Sweeney MD, Chakhoyan A (2020). APOE4 leads to blood–brain barrier dysfunction predicting cognitive decline. Nature.

[287] Riphagen JM, Ramakers IHGM, Freeze WM, Pagen LHG, Hanseeuw BJ, Verbeek MM (2020). Linking APOE-ε4, blood-brain barrier dysfunction, and inflammation to Alzheimer’s pathology. Neurobiol Aging.

[288] Reiss AB, Gulkarov S, Jacob B, Srivastava A, Pinkhasov A, Gomolin IH (2024). Mitochondria in Alzheimer’s Disease Pathogenesis. Life.

[289] Simonovitch S, Schmukler E, Masliah E, Pinkas-Kramarski R, Michaelson DM (2019). The effects of APOE4 on mitochondrial dynamics and proteins in vivo. J Alzheimers Dis.

[290] Hsiao K, Chapman P, Nilsen S, Eckman C, Harigaya Y, Younkin S (1996). Correlative memory deficits, abeta elevation, and amyloid plaques in transgenic mice. Science.

[291] Luo Y, Bolon B, Kahn S, Bennett BD, Babu-Khan S, Denis P (2001). Mice deficient in BACE1, the Alzheimer’s beta-secretase, have normal phenotype and abolished beta-amyloid generation. Nat Neurosci.

[292] Luo Y, Bolon B, Damore MA, Fitzpatrick D, Liu H, Zhang J (2003). BACE1 (β-secretase) knockout mice do not acquire compensatory gene expression changes or develop neural lesions over time. Neurobiol Dis.

[293] Ohno M, Sametsky EA, Younkin LH, Oakley H, Younkin SG, Citron M (2004). BACE1 deficiency rescues memory deficits and cholinergic dysfunction in a mouse model of Alzheimer’s disease. Neuron.

[294] Laird FM, Cai H, Savonenko AV, Farah MH, He K, Melnikova T (2005). BACE1, a major determinant of selective vulnerability of the brain to amyloid-beta amyloidogenesis, is essential for cognitive, emotional, and synaptic functions. J Neurosci.

[295] Ohno M, Cole SL, Yasvoina M, Zhao J, Citron M, Berry R (2007). BACE1 gene deletion prevents neuron loss and memory deficits in 5XFAD APP/PS1 transgenic mice. Neurobiol Dis.

[296] Oakley H, Cole SL, Logan S, Maus E, Shao P, Craft J (2006). Intraneuronal beta-amyloid aggregates, neurodegeneration, and neuron loss in transgenic mice with five familial Alzheimer’s disease mutations: potential factors in amyloid plaque formation. J Neurosci.

[297] Ohno M, Chang L, Tseng W, Oakley H, Citron M, Klein WL (2006). Temporal memory deficits in Alzheimer’s mouse models: rescue by genetic deletion of BACE1. Eur J Neurosci.

[298] Ohno M (2006). Genetic and pharmacological basis for therapeutic inhibition of beta- and gamma-secretases in mouse models of Alzheimer’s memory deficits. Rev Neurosci.

[299] Plant LD, Webster NJ, Boyle JP, Ramsden M, Freir DB, Peers C, Pearson HA (2006). Amyloid beta peptide as a physiological modulator of neuronal ‘A’-type K + current. Neurobiol Aging.

[300] Kamenetz F, Tomita T, Hsieh H, Seabrook G, Borchelt D, Iwatsubo T (2003). APP processing and synaptic function. Neuron.

[301] Singer O, Marr RA, Rockenstein E, Crews L, Coufal NG, Gage FH (2005). Targeting BACE1 with siRNAs ameliorates Alzheimer disease neuropathology in a transgenic model. Nat Neurosci.

[302] McConlogue L, Buttini M, Anderson JP, Brigham EF, Chen KS, Freedman SB (2007). Partial reduction of BACE1 has dramatic effects on Alzheimer plaque and synaptic pathology in APP transgenic mice. J Biol Chem.

[303] Kim T, Vidal GS, Djurisic M, William CM, Birnbaum ME, Garcia KC (2013). Human LilrB2 is a β-amyloid receptor and its murine homolog PirB regulates synaptic plasticity in an Alzheimer’s model. Science.

[304] Amin L, Harris DA (2021). Aβ receptors specifically recognize molecular features displayed by fibril ends and neurotoxic oligomers. Nat Commun.

[305] Dinamarca M, Ríos J, Inestrosa N. Postsynaptic receptors for Amyloid-β oligomers as mediators of neuronal damage in Alzheimer’s Disease. Front Physiol. 2012;3. 10.3389/fphys.2012.00464. 10.3389/fphys.2012.00464 PMC3526732 23267328

[306] Naveilhan P, Neveu I, Baudet C, Funakoshi H, Wion D, Brachet P, Metsis M (1996). 1,25-Dihydroxyvitamin D3 regulates the expression of the low-affinity neurotrophin receptor. Brain Res Mol Brain Res.

[307] Hashimoto Y, Kaneko Y, Tsukamoto E, Frankowski H, Kouyama K, Kita Y (2004). Molecular characterization of neurohybrid cell death induced by Alzheimer’s amyloid-beta peptides via p75NTR/PLAIDD. J Neurochem.

[308] Perini G, Della-Bianca V, Politi V, Della Valle G, Dal-Pra I, Rossi F, Armato U (2002). Role of p75 neurotrophin receptor in the neurotoxicity by beta-amyloid peptides and synergistic effect of inflammatory cytokines. J Exp Med.

[309] Etique N, Verzeaux L, Dedieu S, Emonard H (2013). LRP-1: a checkpoint for the extracellular matrix proteolysis. Biomed Res Int.

[310] Wijnberg MJ, Quax PH, Nieuwenbroek NM, Verheijen JH (1997). The migration of human smooth muscle cells in vitro is mediated by plasminogen activation and can be inhibited by alpha2-macroglobulin receptor associated protein. Thromb Haemost.

[311] Linden R, Martins VR, Prado MA, Cammarota M, Izquierdo I, Brentani RR (2008). Physiology of the prion protein. Physiol Rev.

[312] Ribeiro FM, Paquet M, Cregan SP, Ferguson SS (2010). Group I metabotropic glutamate receptor signalling and its implication in neurological disease. CNS Neurol Disord Drug Targets.

[313] Bordi F, Ugolini A (1999). Group I metabotropic glutamate receptors: implications for brain diseases. Prog Neurobiol.

[314] Wang HY, Stucky A, Liu J, Shen C, Trocme-Thibierge C, Morain P (2009). Dissociating beta-amyloid from alpha 7 nicotinic acetylcholine receptor by a novel therapeutic agent, S 24795, normalizes alpha 7 nicotinic acetylcholine and NMDA receptor function in Alzheimer’s disease brain. J Neurosci.

[315] Olsen KM, Sheng M (2012). NMDA receptors and BAX are essential for Aβ impairment of LTP. Sci Rep.

[316] Zhang H, Jiang X, Ma L, Wei W, Li Z, Chang S (2022). Role of Aβ in Alzheimer’s-related synaptic dysfunction. Front Cell Dev Biol.

[317] Wang D, Govindaiah G, Liu R, De Arcangelis V, Cox CL, Xiang YK (2010). Binding of amyloid beta peptide to beta2 adrenergic receptor induces PKA-dependent AMPA receptor hyperactivity. Faseb j.

[318] Kandouz M (2018). Dying to communicate: apoptotic functions of Eph/Ephrin proteins. Apoptosis.

[319] Sinclair P, Baranova A, Kabbani N. Mitochondrial disruption by amyloid beta 42 identified by proteomics and pathway mapping. Cells 2021: 10, 2380. s Note: MDPI stays neutral with regard to jurisdictional claims in published; 2021. 10.3390/cells10092380 PMC8468661 34572029

[320] Zhong S, Pei D, Shi L, Cui Y, Hong Z (2019). Ephrin-B2 inhibits Aβ(25–35)-induced apoptosis by alleviating endoplasmic reticulum stress and promoting autophagy in HT22 cells. Neurosci Lett.

[321] Kawaguchi Y, Matsubayashi J, Kawakami Y, Nishida R, Kurihara Y, Takei K (2022). LOTUS suppresses amyloid β-induced dendritic spine elimination through the blockade of amyloid β binding to PirB. Mol Med.

